# Construction and Validation of a Macrophage-Associated Risk Model for Predicting the Prognosis of Osteosarcoma

**DOI:** 10.1155/2021/9967954

**Published:** 2021-06-02

**Authors:** Kang-Wen Xiao, Zhi-Bo Liu, Zi-Hang Zeng, Fei-Fei Yan, Ling-Fei Xiao, Jia-Li Li, Lin Cai

**Affiliations:** ^1^Department of Orthopedics, Zhongnan Hospital of Wuhan University, Wuhan, Hubei430071, China; ^2^Department of Radiation and Medical Oncology, Zhongnan Hospital of Wuhan University, Wuhan, Hubei430071, China

## Abstract

**Background:**

Osteosarcoma is one of the most common bone tumors among children. Tumor-associated macrophages have been found to interact with tumor cells, secreting a variety of cytokines about tumor growth, metastasis, and prognosis. This study aimed to identify macrophage-associated genes (MAGs) signatures to predict the prognosis of osteosarcoma.

**Methods:**

Totally 384 MAGs were collected from GSEA software C7: immunologic signature gene sets. Differential gene expression (DGE) analysis was performed between normal bone samples and osteosarcoma samples in GSE99671. Kaplan–Meier survival analysis was performed to identify prognostic MAGs in TARGET-OS. Decision curve analysis (DCA), nomogram, receiver operating characteristic (ROC), and survival curve analysis were further used to assess our risk model. All genes from TARGET-OS were used for gene set enrichment analysis (GSEA). Immune infiltration of osteosarcoma sample was calculated using CIBERSORT and ESTIMATE packages. The independent test data set GSE21257 from gene expression omnibus (GEO) was used to validate our risk model.

**Results:**

5 MAGs (MAP3K5, PML, WDR1, BAMBI, and GNPDA2) were screened based on protein-protein interaction (PPI), DGE, and survival analysis. A novel macrophage-associated risk model was constructed to predict a risk score based on multivariate Cox regression analysis. The high-risk group showed a worse prognosis of osteosarcoma (*p* < 0.001) while the low-risk group had higher immune and stromal scores. The risk score was identified as an independent prognostic factor for osteosarcoma. MAGs model for diagnosis of osteosarcoma had a better net clinical benefit based on DCA. The nomogram and ROC curve also effectively predicted the prognosis of osteosarcoma. Besides, the validation result was consistent with the result of TARGET-OS.

**Conclusions:**

A novel macrophage-associated risk score to differentiate low- and high-risk groups of osteosarcoma was constructed based on integrative bioinformatics analysis. Macrophages might affect the prognosis of osteosarcoma through macrophage differentiation pathways and bring novel sights for the progression and prognosis of osteosarcoma.

## 1. Introduction

Osteosarcoma, as a common malignant tumor, occurred mostly in the distal femur and proximal tibia metaphyses. Currently, the incidence of osteosarcoma worldwide was three to four per million people [1]. The main treatment for osteosarcoma consisted of chemotherapy and surgery [2]. As a highly aggressive tumor, nearly half of osteosarcoma would metastasize, and the lung was the most common metastatic site [3]. Despite a relatively low incidence of osteosarcoma, the prognosis of osteosarcoma continued to be very poor, and the cure rate after surgery was not high (17%) [4]. Hence, it was significant to explore new and effective methods to treat osteosarcoma.

The tumor microenvironment (TME) mainly consisted of tumor cells, extracellular matrix proteins, blood vessels, fibroblasts, immune cells, endothelial cells, and extracellular factors [5]. In the last few decades, TME has been paid more attention and studied in many fields, including tumor angiogenesis and metastasis [6]. Being an important part of the TME, tumor-associated macrophages (TAM) have been observed to affect the metastasis and prognosis of tumors [7]. A recent study showed that the infiltration of macrophages was related to the prognosis of breast cancer through paracrine interaction between breast cancer cells and macrophages [8]. A previous research also revealed that macrophages would promote the growth of epithelial cells with cancer-related mutations [9]. A recent study also implied that TAM prevented metastasis in high-grade osteosarcoma by collecting 132 clinical osteosarcoma samples [10]. TAM could be divided into three types based on their functional properties: M1, M2, and M0 [11]. M1 macrophages, activated by lipopolysaccharides, Th1, and other cytokines, played an important role in promoting inflammation and antimicrobial [12]. Meanwhile, M2 macrophages, mainly induced by CSF-1 and IL-10, were involved in tumor progression, wound healing, tissue repairing, and inhibition of inflammation [13]. It is currently believed that M2 macrophages promoted tumor growth and metastasis while M1 macrophages inhibited tumor formation by secreting cytokines [14]. An injection of M1 macrophages into mice with Ehrlich ascites carcinoma could improve the survival rate of the mice [15]. A former study also indicated that M1 macrophages could reduce the growth of colon cancer cells [16]. Moreover, a high proportion of M2 macrophages could lead to a poor prognosis in ovarian cancer [17]. In Zhou's study, osteosarcoma metastasis could be prevented by all-trans retinoic acid through the prohibition of M2 polarization [18]. Although osteosarcoma and TAM have been studied in recent years, the TAM-related diagnosis and prognostic indicators of osteosarcoma were still rare. Furthermore, most of the current osteosarcoma indicators were based on the hematological study [19], and the influence of the tumor microenvironment on osteosarcoma was rarely considered. Therefore, developing a macrophage-associated risk model to predict the prognosis of osteosarcoma was urgently needed.

Therapeutically applicable research to generate effective treatments (TARGET) was a database of pediatric tumors, including acute lymphoblastic leukemia, acute myeloid leukemia, kidney tumors, neuroblastoma, and osteosarcoma. The CIBERSORT deconvolution algorithm was a machine learning method to calculate the infiltration of various immune cells based on bulk transcriptome data through linear support vector regression and highly robust to noise [20]. LM22 was a gene expression label matrix of immune cells. It consisted of 547 genes that could differentiate 22 immune cell phenotypes [21]. CIBERSORT has been widely used in predicting the proportion of immune cells in cancers. The Connectivity Map (cMap) was an instrumental online bioinformatics database that includes gene expression profiles of more than 7,000 tumor cell lines and 1,309 drugs [22]. Decision curve analysis was a statistical method to evaluate clinical prediction models, diagnostic experiments, and molecular markers [23]. Nomogram could simplify the complex prediction model in the probability estimation of the event (such as death or recurrence) based on the specific situation of each patient. Multivariate Cox regression analysis has been widely applied in clinical research [24]. Here, the clinical and transcriptome data of osteosarcoma from the TARGET database (TARGET-OS) were collected in this study. 384 MAGs were collected from GSEA software C7: immunologic signature gene sets [25, 26]. The STRING database was further used to detect MAGs with a high connection [27]. Then 5 MAGs were selected to construct the risk model through integrative bioinformatics analysis. Moreover, the prognostic nomogram was constructed to evaluate our risk model and further validated by a bootstrap test. Besides, the independent data set from the GEO database was collected to validate our model. In this study, we aimed to identify macrophage-associated gene signatures to predict the prognosis of osteosarcoma, which could help clinicians evaluate patients' conditions and provide novel insights for osteosarcoma.

## 2. Materials and Methods

### 2.1. Sample Collection and Data Preprocessing

TARGET-OS including 88 osteosarcoma samples and clinical information was collected from the TARGET database (https://ocg.cancer.gov/programs/target). Besides, the human genome annotation GTF file was collected from the GENCODE platform (https://www.gencodegenes.org/). Moreover, the test data set GSE21257 consisting of 53 osteosarcoma samples and clinical information was collected from the GEO database (https://www.ncbi.nlm.nih.gov/geo/). TARGET-OS had 88 samples including 50 males and 37 females (1 sample with unknown gender) while GSE21257 consisted of 53 samples including 34 males and 19 females. GSE99671 had 36 samples (22 males and 14 females) including 18 normal bone samples and 18 osteosarcoma samples. The median ages (interquartile range (IQR)) of TARGET-OS and GSE21257 were 14.5 (12.2–17.4) years and 16.7 (13.6–18.7) years, respectively. GPL10295 platform was used for the GSE21257 data set while the GPL20148 platform was used for the GSE99671data set. Robust spline normalization was performed in GSE21257 while normalization of fragments per kilobase of exon model per million mapped fragments (FPKM) was performed in the TARGET-OS data set. Gene sets involving macrophage from GSEA software C7: immunologic signature were selected by searching using the keyword “macrophage.” Then, a total of 384 MAGs were collected after removing overlapped genes. These 384 MAGs were listed in Table S1. All data were normalized through the z-score method:

(1) $$\begin{array}{c} z=\frac{x-\mu }{\sigma }, \end{array}$$

where *Z*was the standard value; *x*was the specific gene expression value; *µ*was the mean expression value of each sample; and *σ*was the standard deviation.

**Figure 1:**
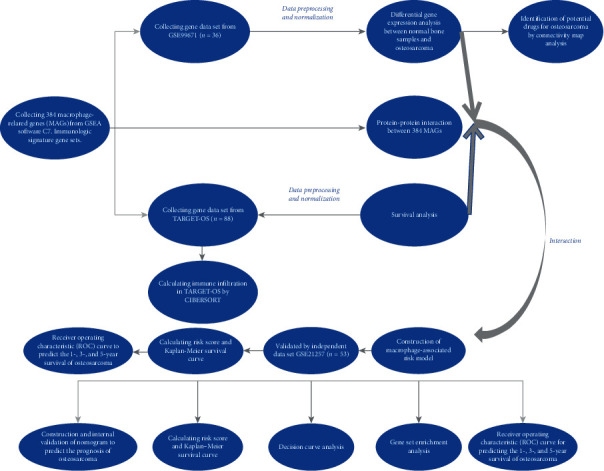
Flowchart of this study.

The probe was a fluorescent-labeled nucleic acid complementary to a specific nucleotide sequence of the target gene. The RNA or cDNA fragment of the gene was captured by base complementary hybridization. Here, we used each probe to match genes. The maximum value of the probe was selected when the gene matched at least two probes. TARGET-OS was the training data set while GSE21257 was the test data set. Detailed information about patients with complete clinical information in TARGET-OS and GSE21257 were presented in Tables 1 and 2. Basic information of three data sets were provided in Table 3. The flowchart of this study was shown in Figure 1.

### 2.2. Differential Gene Expression Analysis and Protein-Protein Interaction Analysis

Totally 384 MAGs were used for DGE using the limma package [28] in R software. Gene signatures of normal bone samples or osteosarcoma were identified based on DGE analysis. Benjamini–Hochberg (BH) method [29] was used here to adjust multiple hypotheses. The screening criteria for significant genes were adjusted to *p* < 0.05. The differentially expressed genes were further transformed into GRP files and uploaded to the cMap database (http://www.broad.mit.edu/cmap/) for small molecule drug prediction analysis. Potential therapeutic targets for osteosarcoma were selected based on enrichment score and *p* < 0.05. Meanwhile, 384 MAGs were used for PPI analysis in the STRING database and further visualized by Cytoscape [30]. The confidence level was 0.4, and MAGs with degrees higher than 2 were selected for further analysis.

### 2.3. Analysis of Infiltration of Immune Cells in Osteosarcoma Samples

The infiltration of immune cells in TARGET-OS was calculated by the CIBERSORT deconvolution algorithm. As a part of the CIBERSORT deconvolution algorithm, the machine learning method, nu-support vector regression (*ν*-SVR), was applied to this analysis. Different *ν* values including 0.25, 0.5, and 0.75 were selected to perform *ν*-SVR to predict the infiltration of immune cells for each sample. The solution of *ν*-SVR was screened based on the lowest root-mean-square error between the true and the predicted expressions. The formula of the CIBERSORT algorithm was as follows:

(2) $$\begin{array}{c} M_{ij}=\sum_{k=1}^{r}S_{ik}F_{cj}, \end{array}$$

where *M*_*ij* _represented the expression level of gene *i* in mixed sample *j*, which was the sum of its expression in *r* immune cell type. *S*_*ik*_was a label matrix (LM22; https://cibersort.stanford.edu/download.php), which represented the gene expression level of gene *i* in immune cells. *F*_*cj*_represented the proportion of cell types in the mixed sample *j*. The permutations of the signature matrix were 1,000. The ESTIMATE package was also used to calculate the immune and stromal scores of each sample [31].

### 2.4. Survival Analysis

The survival and survminer packages were used for survival analysis in R software. The collected 384 MAGs were divided into high- and low-risk groups based on their expression in the TARGET-OS data set for survival analysis. Besides, high- and low-risk groups from the TARGET-OS and the GSE21257 data sets were used for survival analysis, respectively. High/low immune and high/low stromal score groups were also used for survival analysis. The survival rate was compared by the log-rank test. *p* < 0.05 indicated statistical significance.

### 2.5. Construction of Multivariate Cox Regression Model

Multivariate Cox regression model was constructed based on 5 differentially expressed MAGs from the TARGET-OS data set using survival package (https://cran.r-project.org/web/packages/survival/index.html) and survminer package (https://cran.r-project.org/web/packages/survminer/index.html) in R software. For each MAG, the coefficient of multivariate Cox regression was regarded as the coefficients in the risk score. Then the risk score of each sample was calculated. The formula was as follows:

(3) $$\begin{array}{c} \text{risk score}=\sum_{j=1}^{n}{\text{coef}}_{j}{}^{*}X_{j}. \end{array}$$

where coef was the coefficient of multivariate regression analysis of MAGs. *X*was the expression of each MAG. This model was constructed to predict risk scores in osteosarcoma samples. The risk here referred to the risk of a poor prognosis in the osteosarcoma samples. The higher the risk score, the higher the probability of the poor prognosis in osteosarcoma, and vice versa. Osteosarcoma samples were divided into high- and low-risk groups by risk score. Pheatmap package (https://cran.r-project.org/web/packages/pheatmap/index.html) was used to display the expression of MAGs in the high- and low-risk groups in R software. Then risk score and other clinical characteristics were also used for multivariate Cox regression analysis to identify potential independent prognostic factors of osteosarcoma in the TARGET- OS and the GSE21257 data sets.

### 2.6. Decision Curve and ROC Analyses

The DCA curve was shown with the net benefit rate as the ordinate and the high-risk thresholds as the abscissa using the rmda package (https://cran.r-project.org/web/packages/rmda/index.html) in R software. The calculation formula of net benefit was as follows:

(4) $$\begin{array}{c} \text{net benefit}=\frac{\text{true positive}}{n}-\frac{\text{false positive}}{n}{}^{*}\frac{p_{t}}{1-p_{t}}. \end{array}$$

where *n*was the sample size, and *p*_*t*_was the threshold probability. Here, we explored three clinical prognostic models in TARGET-OS: simple clinical data model (gender, race, age, and tumor metastasis), simple MAGs model (5 MAGs and risk score), and complex model that integrated MAGs and clinical features (gender, race, age, tumor metastasis, 5 MAGs, and risk score). The model with the greatest net benefit under different high-risk thresholds was recommended for clinical applications of osteosarcoma. Meanwhile, ROC curves to predict the 1-, 3-, and 5-year survival of TARGET-OS and GSE21257 were performed using the time ROC package in R software [32].

### 2.7. Construction and Internal Validation of Prognostic Nomogram

The nomogram was constructed to predict the overall survival (1 year, 3 years, and 5 years) of patients in TARGET-OS using the rms package (https://cran.r-project.org/web/packages/rms/index.html) in R software. Here, patients' clinical characteristics such as age, gender, tumor metastasis, race, and risk score were used for the construction of a nomogram. Then internal validation of nomogram was performed, and bootstrap was set to 1,000. The discrimination of nomogram was evaluated by concordance index while calibration plots of 1-, 3-, and 5-year survival curves of TARGET-OS were performed to evaluate the prediction performance of nomogram.

### 2.8. Gene Set Enrichment Analysis

All genes from TARGET-OS were used for GSEA based on low- and high-risk groups. Significant macrophage-associated pathways in the osteosarcoma microenvironment were identified using GSEA software. False discovery rate (FDR) < 0.05 and *p* < 0.05 indicated statistical significance.

### 2.9. Statistical Analysis

Statistical Product and Service Solutions software (SPSS 22.0) and R 3.6.2 were used for data analysis. A chi-square test was performed for categorical data. The independent-samples Kruskal–Wallis test was performed to compare MAGs expression between normal bone samples and osteosarcoma samples. Meanwhile, the Kruskal-Walls test was also performed to compare stromal and immune scores between high- and low-risk groups, respectively. All significance levels were *p* < 0.05.

## 3. Results

### 3.1. Identification of 5 MAGs Related to the Prognosis of Osteosarcoma

**Figure 2:**
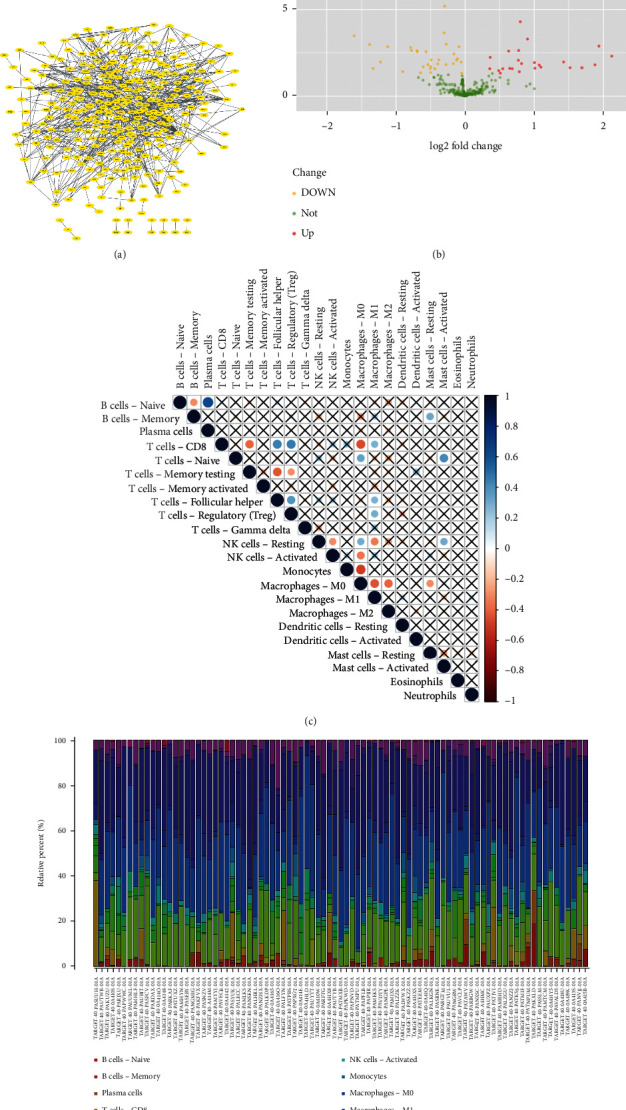
Identification of prognostic MAGs through PPI and DGE and immune infiltration of TARGET-OS: (a) PPI analysis of 384 MAGs, (b) differential gene expression analysis between osteosarcoma samples and normal bone sample, (c) correlation plot of each immune cell in TARGET-OS, and (d) immune infiltration of each sample in TARGET-OS.

**Figure 3:**
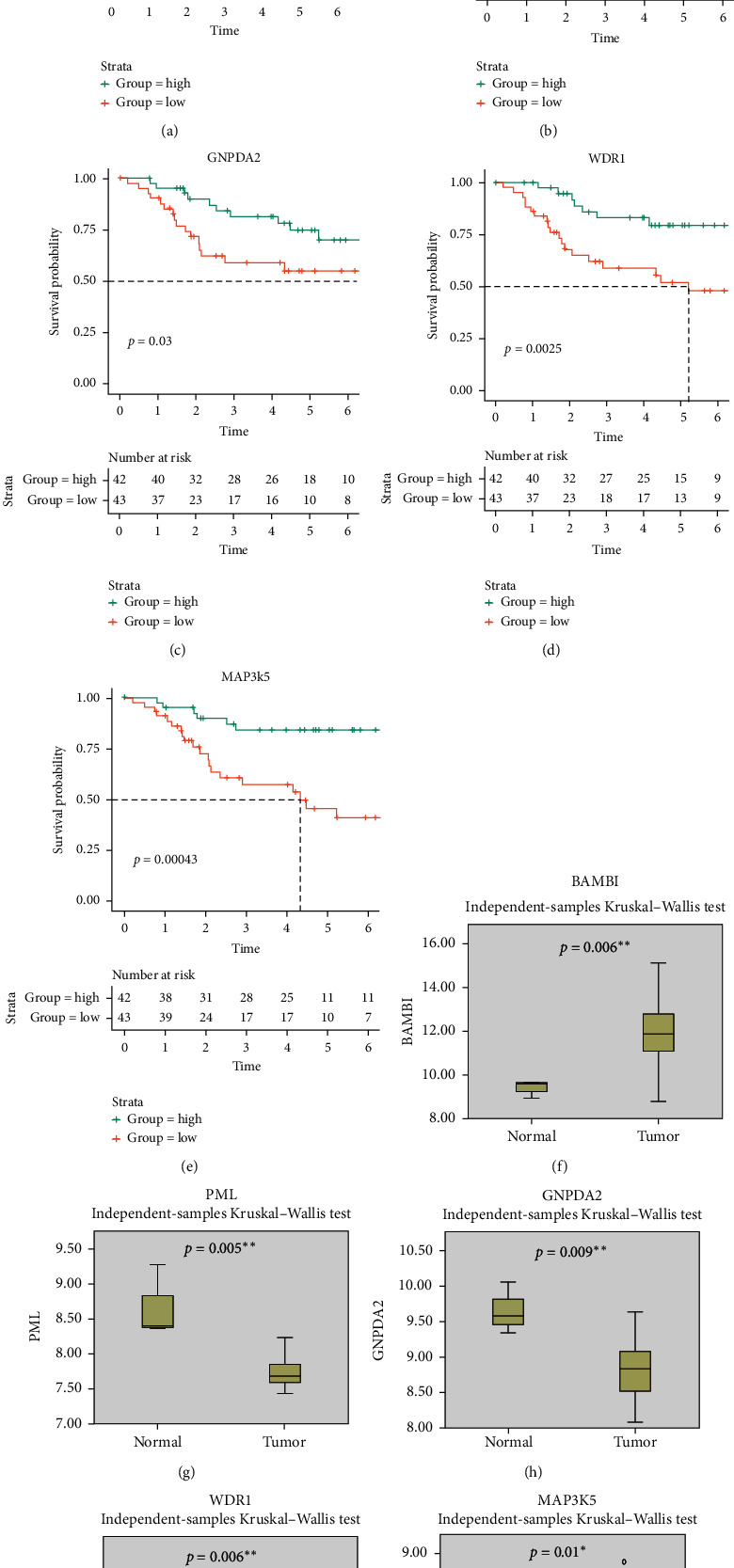
The results of survival curve for 5 MAGs (BAMBI, PML, GNPDA2, WDR1, and MAP3K5) and the expression of each MAG in a normal bone sample and osteosarcoma: (a)–(e) survival curve of 5 MAGs (BAMBI, PML, GNPDA2, WDR1, and MAP3K5) by Kaplan–Meier method and (f)–(j) the boxplots of expression of each MAG in normal bone sample and osteosarcoma. ^*∗*^*p* < 0.05 and ^*∗∗*^*p* < 0.01.

A total of 384 MAGs were screened and used for subsequent analysis. PPI analysis of these 384 MAGs was shown in Figure 2(a). Most MAGs were well-connected to each other. Compared with macrophages in normal bone samples, TAM had different functions and played an important role in tumor progression. Therefore, DGE analysis was used to identify marker MAGs in normal bone samples and osteosarcoma. The result of DGE analysis between normal bone sample and osteosarcoma in the GSE99671 data set was shown in Figure 2(b). Gene BAMBI (*p* = 0.024) and gene ALOX5AP (*p* = 0.001) were top upregulated genes in osteosarcoma while gene WDR1 (*p* = 0.042), gene PML (*p* = 0.012), gene MAP3K5 (*p* = 2.60*E* − 05), gene GNPDA2 (*p* = 0.027), gene CCL5 (*p* = 7.37*E* − 08), and gene MAOA (*p* = 9.08*E* − 07) were top upregulated genes in normal bone sample. cMap analysis was also performed based on these differentially expressed MAGs, and the results were shown in Table 4. Exisulind (*p* = 0.031), atractyloside (*p* = 0.006), and doxycycline (*p* = 0.010) were identified as potential drugs for osteosarcoma. Immune infiltration of each sample in TARGET-OS and the correlation between immune cells were shown in Figures 2(c) and 2(d), respectively. The average proportions of M0, M1, and M2 macrophages were 43.4%, 2.35%, and 27.2%, respectively. The median proportions (IQR) of M0, M1, and M2 macrophages were 46.6% (32.2%–54.3%), 1.36% (0.420%–3.41%), and 23.4% (18.5%–36.8%), respectively. Moreover, Kaplan–Meier survival analysis of 384 MAGs in TARGET-OS was performed. Then a total of 5 intersection MAGs (WDR1, PML, MAP3K5, GNPDA2, and BAMBI) were screened based on the results of DGE, PPI, and survival analysis. The influence of different gene expression levels on the prognosis of osteosarcoma was further explored. The survival curve of each MAG in TARGET-OS was shown in Figures 3(a)–3(e). Gene WDR1 (*p* = 0.003), PML (*p* = 0.002), MAP3K5 (*p* < 0.001), and GNPDA2 (*p* = 0.030) showed a better prognosis of osteosarcoma in the high expression group. Moreover, gene BAMBI in the high expression group showed a worse prognosis (*p* = 0.013). Besides, the relative expression of each MAG in normal bone samples and osteosarcoma sampleswas shown in Figures 3(f)–3(j). The expression of gene WDR1 (*p* = 0.006), PML (*p* = 0.005), MAP3K5 (*p* = 0.010), and GNPDA2 (*p* = 0.009) were significantly higher in normal bone sample while in osteosarcoma samples, the expression of BAMBI (*p* = 0.006) was significantly higher. Then the effect of different expression levels of these 5 MAGs on gender and age was also explored. However, the results turned out that these 5 MAGs were not significantly related to gender or age. These 5 MAGs were used for subsequent analysis.

### 3.2. Construction of a Macrophage-Associated Risk Model

The macrophage-associated risk model was constructed by 5 MAGs through multivariate Cox regression analysis in TARGET-OS. The formula of our risk model was as follows:

(5) $$\begin{array}{c} \text{risk score}=\left(-0.767\right){}^{*}\text{WDR}1+\left(-0.674\right){}^{*}\text{PML}+\left(-2.046\right){}^{*}\text{MAP}3\text{K}5+\left(-2.534\right){}^{*}\text{GNPDA}2+\left(-0.307\right){}^{*}\text{BAMBI.} \end{array}$$

**Figure 4:**
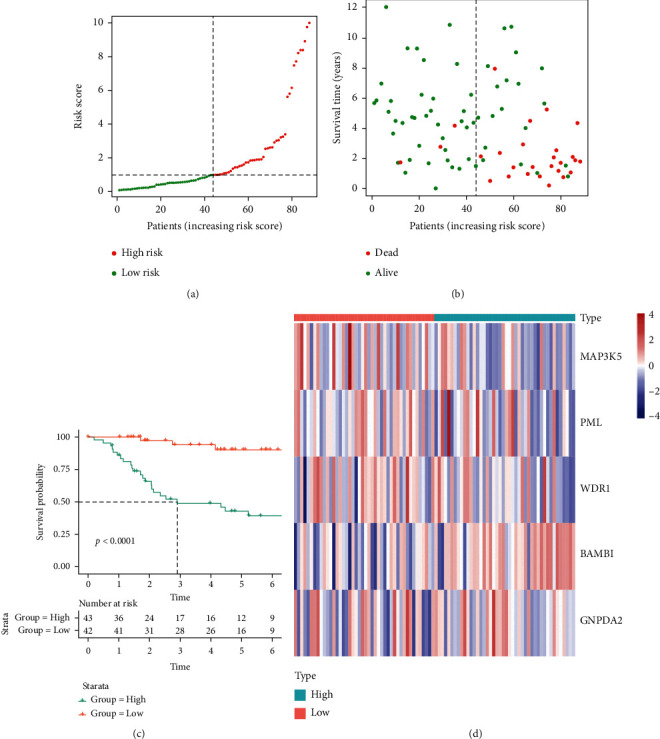
Construction of macrophage-associated risk model and survival curve of high-/low-risk groups in TARGET-OS: (a) TARGET-OS was divided into high- and low-risk groups using the median risk score as the cutoff value, (b) the relationship between risk score and survival time and status of patients, (c) the survival curve of high- and low-risk groups in TARGET-OS, and (d) the heat map between expression of 5 MAGs and osteosarcoma samples.

**Figure 5:**
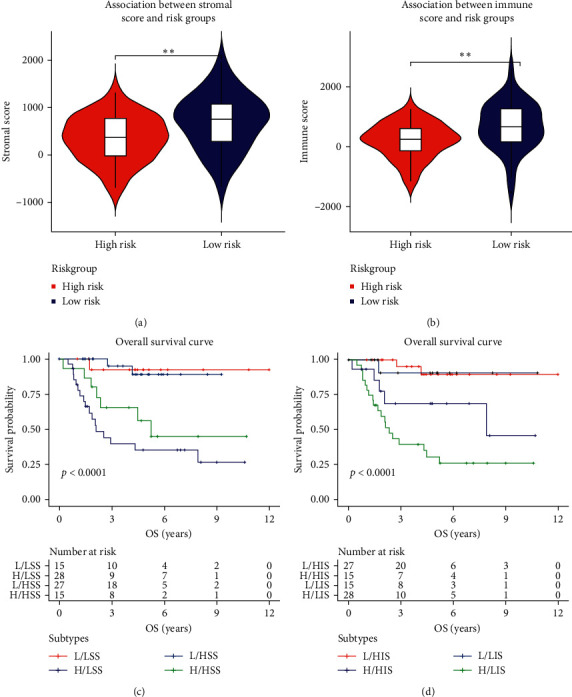
Comparison of stromal and immune scores among high- and low-risk groups and survival analysis: (a) comparison of stromal score among high- and low-risk groups, (b) comparison of immune score among high- and low-risk groups, (c) survival analysis of different stromal scores among high- and low-risk groups (L: low, H: high, LSS: low stromal score, and HSS: high stromal score), and (d) survival analysis of different immune scores among high- and low-risk groups (L: low, H: high, LIS: low immune score, and HIS: high immune score).^*∗*^*p* < 0.05and ^*∗∗*^*p* < 0.01.

Then, osteosarcoma samples were divided into high- and low-risk groups by risk score, and the results were shown in Figures 4(a) and 4(b). As the risk score of patients increased, the number of deaths rose, and the survival time of patients decreased. Besides, our score could effectively predict the prognosis of osteosarcoma (concordance index = 0.797). Therefore, in order to further explore the prognostic difference between the high- and low-risk groups, clinical information of high- and low-risk groups were used as training data for survival curve analysis. The result was shown in Figure 4(c). Compared with the high-risk group, the low-risk group had a significantly improved prognosis (*p* < 0.001). The 5-year survival rates of high- and low-risk groups were 43.0% and 90.1%, respectively. A heat map of the expression of 5 MAGs in TARGET-OS was displayed in Figure 4(d). Besides, different stromal and immune scores among high- and low-risk groups were explored. The results turned out that the stromal and immune scores of the low-risk group were both significantly higher than the high-risk group (Figures 5(a) and 5(b)). The survival plot of each group with different immune and stromal scores was subsequently shown in Figures 5(c) and 5(d). The low-risk group continued to have a significantly better prognosis (*p* < 0.0001), regardless of its immune and stromal scores. Since there was a significant prognostic difference between high- and low-risk groups, our risk score was considered to predict the prognosis of osteosarcoma effectively.

### 3.3. Macrophage-Associated Risk Score: An Independent Prognostic Factor of Osteosarcoma

**Figure 6:**
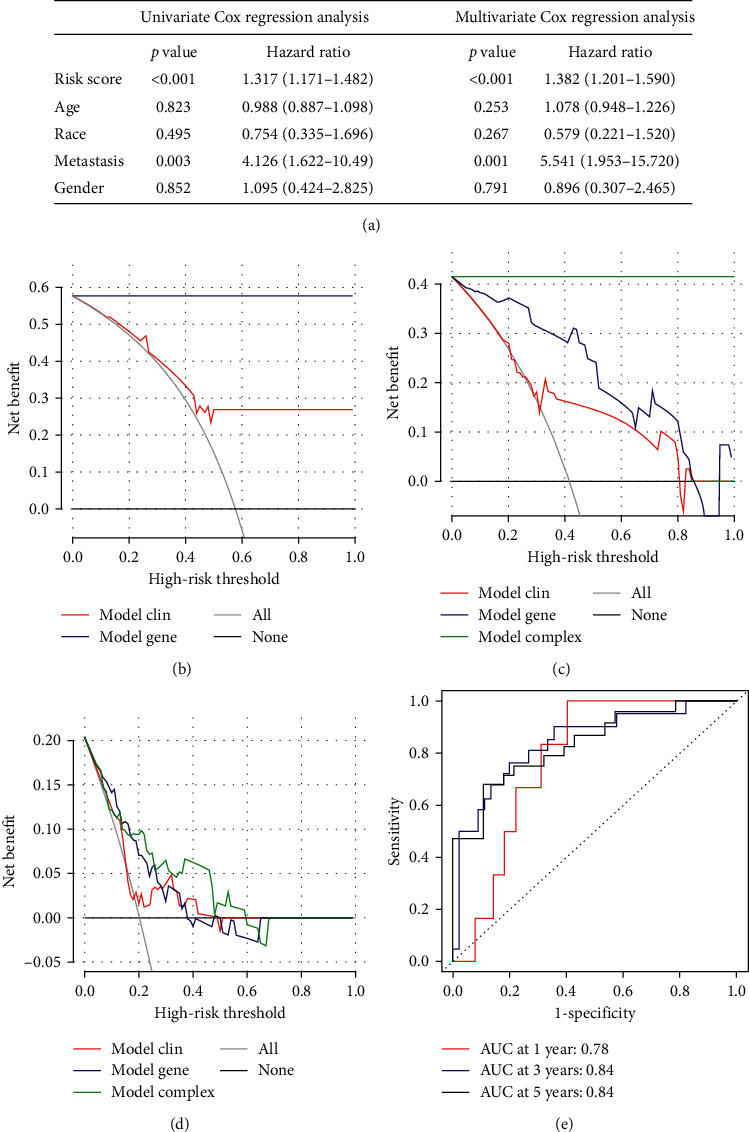
Identification of risk score as an independent prognostic factor of osteosarcoma, decision curve analysis of three models (simple gene model, simple clinical model, and complex model), and ROC curve for predicting the prognosis of osteosarcoma in 1, 3, and 5 years: (a) identification of risk score as an independent prognostic factor of osteosarcoma by univariate and multivariate Cox regression analyses, (b) the decision curve of the net benefit of the 2 models for the 3-year survival rate (simple gene and simple clinical models), (c) the decision curve of the net benefit of the 3 models for the 5-year survival rate (simple gene, simple clinical, and complex models); (d) the decision curve of the net benefit of the 3 models for the diagnosis of osteosarcoma metastasis (simple gene, simple clinical, and complex models), and (e) ROC curve to predict the prognosis of osteosarcoma in 1, 3, and 5 years.

Clinical characteristics might be correlated with the prognosis of osteosarcoma. To explore whether our risk model could independently predict the prognosis, the risk score and other clinical information of TARGET-OS including gender, tumor metastatic, race, and age were used for Cox regression analysis. The results were shown in Figure 6(a). In univariate Cox regression analysis, risk score (*p* < 0.001) and tumor metastatic (*p* = 0.003) were closely related to the prognosis of osteosarcoma. Moreover, in multivariate Cox regression analysis, risk score (*p* < 0.001) and tumor metastatic (*p* = 0.001) were still related to the prognosis of osteosarcoma, indicating that tumor metastatic and our risk score could be considered independent prognostic factors of osteosarcoma.

### 3.4. MAG Model for Diagnosis of Osteosarcoma Had a Better Net Clinical Benefit than the Simple Clinical Model

The decision curve of three clinical prognostic models (simple clinical data model (gender, race, age, and tumor metastasis), simple MAGs model (5 MAGs and risk score), and complex model) in TARGET-OS were shown in Figures 6(b)–6(d). Compared with clinical models, our MAGs model had a better net benefit for patients' 3- and 5-year survival rate (Figures 6(b) and 6(c)). As shown in Figure 6(d), compared with clinical models, our MAGs model had the greatest net benefit to diagnose osteosarcoma metastasis. Among them, the simple clinical data model exhibited the lowest net benefit while the complex model had the highest net benefit. Therefore, a comprehensive analysis of clinical and genetic information could increase the net benefit of the model. Besides, in Figure 6(e), the area under the curve for prediction of 1-, 3-, and 5-year survival of osteosarcoma was 0.78, 0.84, and 0.84, respectively. This result also indicated that our MAGs model could accurately predict the prognosis of osteosarcoma.

### 3.5. Nomogram Effectively Predicted the Prognosis of Osteosarcoma

**Figure 7:**
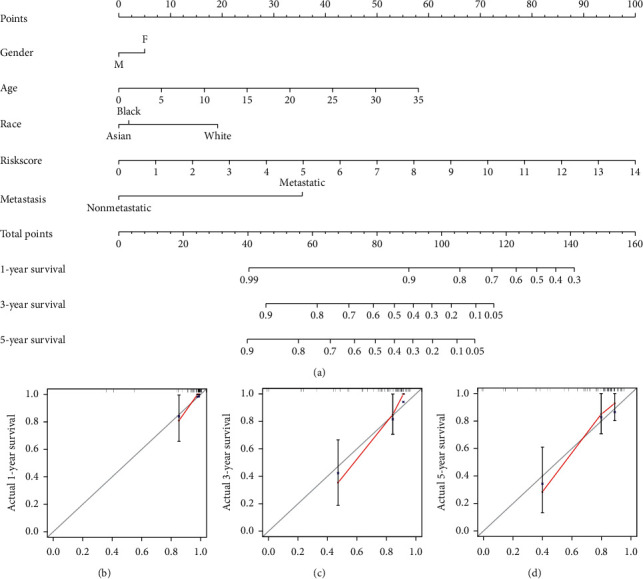
Construction and internal validation of nomogram to predict the prognosis of osteosarcoma: (a) construction of the nomogram by collecting age, gender, tumor metastasis, race, and risk score; (b) 1-year survival calibration curve; (c) 3-year survival calibration curve; and (d) 5-year survival calibration curve.

Since considering both clinical features and our MAGs model had the best net clinical benefit, the nomogram was constructed by integrating gender, age, tumor metastasis, race, and risk score. The result was displayed in Figure 7(a). As the total points became higher, the 1-, 3-, and 5-year survival rate of patients decreased. The concordance index of the nomogram was 0.842, and the calibration curve of 1-, 3-, and 5-year survival of osteosarcoma (Figures 7(b)–7(d)) also illustrated our nomogram that could effectively predict the prognosis of osteosarcoma.

### 3.6. Validation of Our Risk Model by Independent Data Set

**Figure 8:**
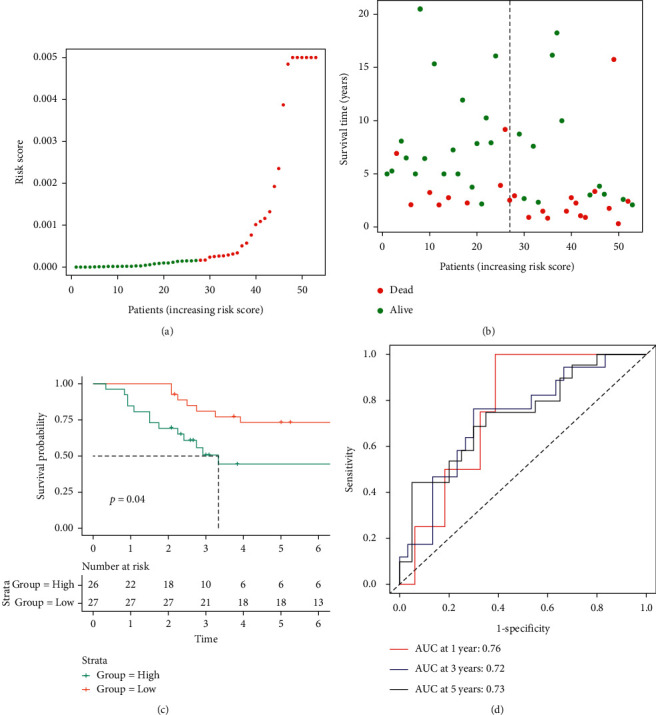
Validation of macrophage-associated risk model and survival curve of high-/low-risk groups in GSE21257: (a) GSE21257 was divided into high- and low-risk groups using the median risk score as the cutoff value, (b) the relationship between risk score and survival time and status of patients, (c) the survival curve of high- and low-risk groups in GSE21257, and (d) ROC curve to predict the prognosis of osteosarcoma in 1, 3, and 5 years.

**Figure 9:**
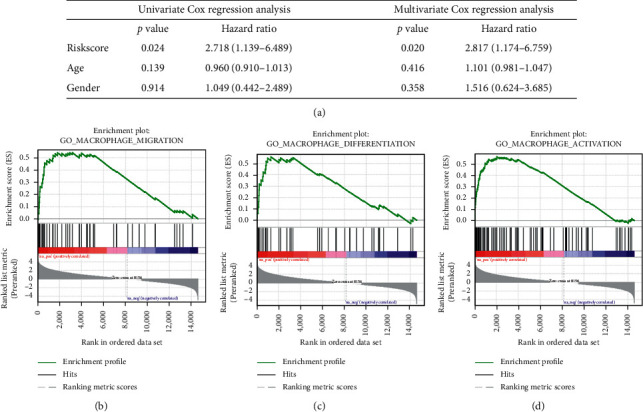
Validation of risk score as an independent prognostic factor of osteosarcoma in GSE21257 and important pathways identified by GSEA: (a) validation of risk score as an independent prognostic factor of osteosarcoma in GSE21257 by univariate and multivariate Cox regression analyses, (b) the important enrichment pathway: GO – macrophage migration, (c) the important enrichment pathway: GO-macrophage differentiation, and (d) the important enrichment pathways: GO-macrophage activation.

The independent data set GSE21257 was used for the validation of our risk model. The GSE21257 data set was divided into high- and low-risk groups based on the macrophage-associated risk model. As shown in Figures 8(a) and 8(b), compared with the low-risk group, the high-risk group had a lower survival time. The survival curve of different risk groups was shown in Figure 8(c). The 5-year survival rates of the high- and low-risk groups were 48.1% and 76.9%, respectively. The low-risk group had better clinical outcome (*p* = 0.040). The areas under the curve for prediction of 1-, 3-, and 5-year survival of osteosarcoma were 0.76, 0.72, and 0.73, respectively (Figure 8(d)). Moreover, the risk score and clinical information of GSE21257 including gender and age were also used for Cox regression analysis. The risk score was significantly correlated with the prognosis of osteosarcoma in univariate (*p* = 0.024) and multivariate (*p* = 0.020) regression analyses (Figure 9(a)). Therefore, our risk score could be considered an independent prognostic factor of osteosarcoma.

### 3.7. Low-Risk Group Was Related to Macrophage Differentiation Pathway

GSEA was also performed for all genes from TARGET-OS. The results were shown in Table S2, and important pathways are shown in Figures 9(b)–9(d). Genes were enriched in GO: macrophage activation (enrichment score (ES) = 0.56, *p* < 0.001, and FDR < 0.001), GO: macrophage migration (ES = 0.54, *p* = 0.001, and FDR = 0.003), GO: macrophage differentiation (ES = 0.56, *p* = 0.001, and FDR = 0.004), and GO: regulation of macrophage chemotaxis (ES = 0.59, *p* = 0.004, and FDR = 0.009). These biological processes were considered important pathways of TAM in the osteosarcoma microenvironment. The above analysis revealed that MAGs played a significant role in osteosarcoma. Moreover, the results of characteristics of clinical information and prognosis of osteosarcoma were consistent with the result of TARGET-OS. Therefore, our risk model could differentiate different risk groups successfully, and the low-risk group was correlated with a better prognosis of osteosarcoma.

## 4. Discussion

As osteosarcoma is one of the most common childhood tumors in the world, its treatment and prognosis have received widespread attention. Despite the surgical treatment along with pre- and postoperative chemotherapy, the event-free 5-year survival rate remained low [33]. A previous study revealed that gene PPARG, gene IGHG3, and gene PDK1 were correlated with osteosarcoma [34]. However, this conclusion was not validated by the independent data set. In this study, DGE analysis was performed based on normal bone samples and osteosarcoma samples to identify differentially expressed MAGs. We further identified exisulind, atractyloside, and doxycycline as top potential drugs for the treatment of osteosarcoma. A recent study illustrated that exisulind could induce apoptosis in lung cancer by downregulating cyclic GMP phosphodiesterase and further improve the prognosis of lung cancer [35]. Atractyloside was also found to suppress the progression and metastasis of colon cancer in Lu's study [36]. A previous report also showed that doxycycline could reduce the proliferation of melanoma cells by inhibiting apoptosis [36]. Considering the important role these drugs played in other tumors, they might become promising drugs for osteosarcoma treatment. The selected 5 MAGs (MAP3K5, PML, WDR1, BAMBI, and GNPDA2) were identified as prognostic-related genes for osteosarcoma, and a novel macrophage-associated risk model was constructed based on these 5 MAGs. MAP3K5, also called ASK1, was an important kinase in the process of cell apoptosis, participating in the regulation of multiple cell-signaling pathways in inflammation and tumors [37]. A recent research indicated that MAP3K5 promoted macrophage activation and migration in mice models [38]. Furthermore, knockout MAP3K5 mice were more likely to develop colon cancer [39]. These research works were also consistent with our study. MAP3K5 might be related to a better prognosis of a tumor by activating macrophages, which also needed further study to validate. The biological function of the gene PML was associated with its nuclear location, and PML was associated with cell cycle regulation and tumor suppression [40]. For instance, reduced expression of PML was found to promote multiple tumor growth, including prostate adenocarcinoma, breast carcinoma, and thyroid carcinoma [41]. A previous research reported that PML was critical to the formation of macrophages [42]. Here, we reported that high expression of PML might inhibit osteosarcoma growth, which was also consistent with these findings. WDR1 took part in inducing the disassembly of actin filaments, and dysfunction of WDR1 might cause autoinflammatory disease, which in turn activated macrophage [43]. BAMBI, a pseudoreceptor of the TGF signaling pathway, played a key role in regulating macrophage polarization [44]. A former study indicated that highly expressed BAMBI could contribute to colon cancer metastasis through Wnt/beta-catenin in mice models [45]. Besides, the expression of BAMBI was significantly higher in ovarian cancer through TGF-beta signaling [46]. BAMBI was also highly expressed in pancreatic cancer by the TGF-beta pathway [47]. Similarly, our study reported that high expression of BAMBI would result in a poor prognosis of osteosarcoma, which implied that BAMBI could be a new target for the treatment of osteosarcoma. GNPDA2 was closely related to obesity and body mass index. A recent epidemiological survey also showed that people with a high body mass index were at higher risk of cancer [48]. Besides, macrophage accumulating in adipose tissue was related to obesity [49]. Therefore, these genes were considered to be potential targets for the treatment of osteosarcoma, and further experiments are needed to verify the role of these genes in osteosarcoma.

Our study showed that the low-risk group had significantly higher immune and stromal scores, which was correlated with a better prognosis. Similarly, a previous study indicated that osteosarcoma samples with high immune scores had a better prognosis [34]. ROC curve also exhibited excellent accuracy of our risk model in TARGET-OS and GSE21257. The decision curve considered the clinical utility of specific models and focused on the net benefit of different clinical interventions. Here, the decision curve of three models (simple gene model, simple clinical model, and complex model) demonstrated that comprehensive consideration of genetic information and clinical information could obtain the greatest benefits. Besides, the nomogram also accurately predicted the prognosis of osteosarcoma. Therefore, our risk model could effectively predict the prognosis of osteosarcoma, which could be a potential clinical indicator of osteosarcoma. The GSEA results of TARGET-OS showed that GO: macrophage activation, GO: macrophage migration, GO: macrophage differentiation, and GO: regulation of macrophage chemotaxis were considered key pathways in osteosarcoma. Macrophage activation and migration were correlated with tumor growth. A recent study reported that activated TAM could promote the angiogenesis of breast cancer [50]. TAM also promoted cancer development by upregulation of LAMP2a [51]. The migration of macrophages was inhibited under hypoxia, which was conducive to tumor growth [52]. Besides, macrophage migration could accelerate tumor invasion without relying on matrix metalloproteinase [53]. Macrophage differentiation also played a significant role in the tumor microenvironment. A previous study showed that M2 macrophage was closely associated with malignant oral squamous cell carcinomas [54]. Macrophage could be shifted to M1 macrophage by phenelzine in triple-negative breast cancer [55]. Moreover, the chemokine system was found to affect macrophage polarization. A former research reported that interferon gamma could induce the activation of M1 macrophage [56]. These biological processes were closely related to TAM in tumor, which also provided a novel research perspective for the role of TAM in osteosarcoma.

Our study, however, had some limitations: (1) due to the limitation of sample size, we needed more research to support our conclusion, and (2) we also lacked prospective clinical trials to verify the performance of our model further. Therefore, we looked forward to more research on MAGs to explore novel ideas for the clinical treatment of osteosarcoma.

## 5. Conclusion

In general, 5 MAGs (MAP3K5, PML, WDR1, BAMBI, and GNPDA2) correlated with the prognosis of osteosarcoma were screened for the construction of the risk model. A novel macrophage-associated risk score to differentiate low- and high-risk groups of osteosarcoma was constructed based on multiple bioinformatics analyses. The high score indicated the poor prognosis of osteosarcoma while the low score indicated the better prognosis of osteosarcoma. Besides, our risk score was validated by the independent data set successfully, and nomogram effectively predicted the prognosis of osteosarcoma.

## Figures and Tables

**Table 1:** Detailed clinical information about the TARGET-OS data set.

Characteristics	Risk group		*p* value
	Low-risk group	High-risk group	
Age (IQR)	15.67 (12.36–16.49)	13.35 (10.08–16.23)	0.173
Gender			
Female	15	12	0.311
Male	14	19	
Race			
White	26	25	0.384
Black	5	2	
Asian	2	4	
Tumor metastasis			
Metastatic	4	8	0.161
Nonmetastatic	29	23	

IQR: interquartile range.

**Table 2:** Detailed clinical information about the GSE21257 data set.

Characteristics	Risk group		*p* value
	Low-risk group	High-risk group	
Age (IQR)	16.67(14.59–18.59)	15.08(13–18.17)	0.258
Gender			
Female	10	9	0.854
Male	17	17	

IQR: interquartile range.

**Table 3:** Detailed information about the GSE21257, the GSE99671, and the TARGET-OS data sets.

	TARGET-OS	GSE21257	GSE99671
Osteosarcoma samples	88	53	36
Male	50	34	22
Female	37	19	14
Research object	Human	Human	Human
Time of uploading chip	Aug 17,2019	Mar 22,2012	Nov 03, 2017
Median ages (IQR)	14.5(12.2–17.4)	16.7(13.6–18.7)	NA

IQR: interquartile range, NA: not applicable.

**Table 4:** Potential drugs for the treatment of osteosarcoma by cMap analysis.

cMap name	Mean	*n*	Enrichment	*p*	Specificity	Percent nonnull
Exisulind	−0.516	2	−0.874	0.03167	0.0287	100
Chenodeoxycholic acid	−0.549	4	−0.698	0.01751	0.1	75
Atractyloside	−0.445	5	−0.695	0.00581	0.0227	60
Clorsulon	−0.409	4	−0.687	0.02077	0.0567	50
Doxycycline	−0.391	5	−0.664	0.01007	0.0226	60
Paromomycin	−0.515	4	−0.654	0.03296	0	75
Naltrexone	−0.352	5	−0.643	0.01448	0.0562	60
Chlormezanone	−0.426	4	−0.641	0.03975	0.0276	75
Indometacin	0.165	8	0.508	0.01946	0.0156	50
Flufenamic acid	0.305	6	0.527	0.04479	0.0511	50
Prilocaine	0.31	6	0.531	0.04177	0.0081	50
Orphenadrine	0.455	6	0.561	0.02674	0.0461	66
Hydralazine	0.259	6	0.607	0.0114	0	50

## Data Availability

The data sets (TARGET-OS, GSE21257, and GSE99671) analyzed during the current study are available in the GEO database (https://www.ncbi.nlm.nih.gov/) and TARGET database (https://ocg.cancer.gov/programs/target).

## Abbreviations

MAG: Macrophage-associated gene
GSEA: Gene set enrichment analysis
DGE: Differential gene expression
PPI: Protein-protein interaction
cMap: Connectivity Map
ROC: Receiver operating characteristic
GEO: Gene expression omnibus
TME: Tumor microenvironment
TAM: Tumor-associated macrophage
TARGET: Therapeutically applicable research to generate effective treatment
FDR: False discovery rate
DCA: Decision curve analysis
ES: Enrichment score.

## Supplementary Materials

Supplementary Materials
*Table S1.* Full list of 384 macrophage-associated genes. *Table S2.* Enrichment analysis in TARGET-OS.
