# Supplementary material for: Construction and Validation of a Macrophage-Associated Risk Model for Predicting the Prognosis of Osteosarcoma
Source: J Oncol. 2021 Jun 2;2021:9967954. doi: 10.1155/2021/9967954 (PMC8192206; doi:10.1155/2021/9967954)
Supplement: Supplementary Materials — Table S1. Full list of 384 macrophage-associated genes. Table S2. Enrichment analysis in TARGET-OS. [file 9967954.f1.zip › 9967954.f1/Table S2.pdf]

NAME	SIZE	ES	NES	NOM p-val	FDR q-val
GO_LYMPHOCYTE_MIGRATION	100	0.66 2682	2.60 5554	0	0
GO_POSITIVE_REGULATION_OF_LEUKOCYTE_CELL_CELL_ADHESION	213	0.59 4902	2.50 326	0	0
GO_REGULATION_OF_T_CELL_ACTIVATION	295	0.57 8123	2.49 9535	0	0
GO_T_CELL_MIGRATION	60	0.68 761	2.49 3484	0	0
GO_POSITIVE_REGULATION_OF_CELL_ACTIVATION	306	0.57 4885	2.48 5302	0	0
GO_T_CELL_SELECTION	46	0.71 8135	2.48 3788	0	0
GO_POSITIVE_REGULATION_OF_CELL_CELL_ADHESION	251	0.57 7862	2.47 1592	0	0
GO_LEUKOCYTE_CELL_CELL_ADHESION	315	0.56 7109	2.46 7614	0	0
GO_REGULATION_OF_ANTIGEN_PROCESSING_AND_PRESENTATION	20	0.84 115	2.45 0723	0	0
GO_POSITIVE_REGULATION_OF_LYMPHOCYTE_ACTIVATION	256	0.57 282	2.44 6987	0	0
GO_T_CELL_PROLIFERATION	173	0.58 8115	2.43 6239	0	0
GO_GRANULOCYTE_MIGRATION	131	0.60 1154	2.42 8709	0	0
GO_LEUKOCYTE_CHEMOTAXIS	192	0.57 1646	2.42 5486	0	0
GO_LYMPHOCYTE_CHEMOTAXIS	55	0.68 3626	2.42 2843	0	0
GO_NEUTROPHIL_MIGRATION	109	0.60 9793	2.41 9596	0	0
GO_POSITIVE_REGULATION_OF_T_CELL_PROLIFERATION	91	0.62 4127	2.41 0845	0	0
GO_PHAGOCYTOSIS	245	0.56 2916	2.40 9646	0	0
GO_NEUTROPHIL_CHEMOTAXIS	91	0.62 8903	2.39 2467	0	0
GO_GRANULOCYTE_CHEMOTAXIS	109	0.60 9363	2.39 2123	0	0
GO_REGULATION_OF_ANTIGEN_RECEPTOR_MEDIATED_SIGNALING_PATHWAY	58	0.66 0735	2.39 1574	0	0
GO_REGULATION_OF_LYMPHOCYTE_ACTIVATION	389	0.54 0677	2.38 4926	0	0

GO_NEGATIVE_REGULATION_OF_ANTIGEN_RECEPTOR_MEDIATED_SIGNALING_PATHWAY	29	0.75 7873	2.38 3657	0	0
GO_NEGATIVE_REGULATION_OF_T_CELL_PROLIFERATION	54	0.66 1625	2.37 9403	0	0
GO_T_CELL_ACTIVATION	428	0.53 7028	2.37 4717	0	0
GO_ADAPTIVE_IMMUNE_RESPONSE	373	0.53 8104	2.35 8241	0	0
GO_T_CELL_ACTIVATION_INVOLVED_IN_IMMUNE_RESPONSE	96	0.60 517	2.35 7699	0	0
GO_POSITIVE_REGULATION_OF_LYMPHOCYTE_MIGRATION	37	0.70 7383	2.34 5374	0	0
GO_POSITIVE_T_CELL_SELECTION	33	0.72 2144	2.34 4931	0	0
GO_REGULATION_OF_T_CELL_MIGRATION	41	0.68 6666	2.34 2113	0	0
GO_DENDRITIC_CELL_DIFFERENTIATION	39	0.69 3192	2.34 0819	0	0
GO_MYELOID_LEUKOCYTE_MIGRATION	189	0.55 3928	2.32 8297	0	0
GO_REGULATION_OF_CELL_ACTIVATION	499	0.52 5062	2.32 645	0	0
GO_REGULATION_OF_CELL_CELL_ADHESION	379	0.52 8376	2.32 3718	0	0
GO_RESPONSE_TO_INTERFERON_GAMMA	184	0.55 9983	2.31 7679	0	0
GO_POSITIVE_REGULATION_OF_T_CELL_MIGRATION	30	0.72 9278	2.31 4566	0	0
GO_POSITIVE_REGULATION_OF_LEUKOCYTE_DIFFERENTIATION	142	0.56 886	2.31 436	0	0
GO_INTERLEUKIN_10_PRODUCTION	50	0.65 9351	2.30 5205	0	0
GO_NEGATIVE_REGULATION_OF_LYMPHOCYTE_ACTIVATION	131	0.56 7592	2.30 2895	0	0
GO_REGULATION_OF_LEUKOCYTE_PROLIFERATION	214	0.54 1933	2.30 2565	0	0
GO_NEGATIVE_REGULATION_OF_LEUKOCYTE_CELL_CELL_ADHESION	107	0.58 1071	2.30 1881	0	0
GO_TUMOR_NECROSIS_FACTOR_BIOSYNTHETIC_PROCESS	34	0.70 1455	2.28 8693	0	0
GO_POSITIVE_REGULATION_OF_LYMPHOCYTE_DIFFERENTIATION	95	0.59 2176	2.28 812	0	0
GO_REGULATION_OF_LYMPHOCYTE_MIGRATION	56	0.63 2686	2.28 5851	0	0

GO_LYMPHOCYTE_ACTIVATION_INVOLVED_IN_IMMUNE_RESPONSE	162	0.55 1658	2.28 3278	0	0
GO_B_CELL_RECEPTOR_SIGNALING_PATHWAY	55	0.63 1155	2.28 0671	0	0
GO_POSITIVE_REGULATION_OF_LEUKOCYTE_MIGRATION	125	0.56 0603	2.27 1138	0	0
GO_REGULATION_OF_LEUKOCYTE_CHEMOTAXIS	102	0.57 7891	2.26 6589	0	0
GO_NEGATIVE_REGULATION_OF_LEUKOCYTE_PROLIFERATION	76	0.60 0802	2.26 6166	0	0
GO_CYTOKINE_METABOLIC_PROCESS	65	0.61 5987	2.26 5264	0	0
GO_POSITIVE_REGULATION_OF_LEUKOCYTE_CHEMOTAXIS	81	0.59 9358	2.26 2508	0	0
GO_REGULATORY_T_CELL_DIFFERENTIATION	30	0.72 0113	2.26 2024	0	0
GO_LEUKOCYTE_MIGRATION	403	0.51 3643	2.25 9166	0	0
GO_MYELOID_LEUKOCYTE_MEDIATED_IMMUNITY	487	0.50 8336	2.25 0419	0	0
GO_RESPIRATORY_BURST	29	0.71 5101	2.24 9501	0	0
GO_LYMPHOCYTE_DIFFERENTIATION	331	0.51 2976	2.24 7431	0	1.84 E-05
GO_INFLAMMATORY_RESPONSE_TO_ANTIGENIC_STIMULUS	48	0.64 5039	2.24 6182	0	1.80 E-05
GO_INTERFERON_GAMMA_PRODUCTION	103	0.56 9137	2.24 3495	0	1.77 E-05
GO_LEUKOCYTE_PROLIFERATION	278	0.51 81	2.23 8519	0	1.74 E-05
GO_REGULATION_OF_DEFENSE_RESPONSE_TO_VIRUS_BY_VIRUS	29	0.70 7042	2.23 1097	0	1.71 E-05
GO_LYMPHOCYTE_MEDIATED_IMMUNITY	233	0.52 3689	2.23 0246	0	1.68 E-05
GO_EOSINOPHIL_MIGRATION	22	0.74 9464	2.22 7357	0	1.66 E-05
GO_POSITIVE_REGULATION_OF_LEUKOCYTE_PROLIFERATION	135	0.54 6517	2.22 6928	0	1.63 E-05
GO_MONOCYTE_CHEMOTAXIS	55	0.62 0264	2.22 4015	0	1.60 E-05
GO_LYMPHOCYTE_COSTIMULATION	57	0.61 9028	2.22 2101	0	1.58 E-05
GO_POSITIVE_REGULATION_OF_CELL_ADHESION	385	0.50 26	2.21 9594	0	3.08 E-05

GO_REGULATION_OF_T_CELL_RECEPTOR_SIGNALING_PATHWAY	39	0.66<br>239	2.21<br>625	0	3.04<br>E-05
GO_REGULATION_OF_LEUKOCYTE_DIFFERENTIATION	255	0.51<br>8404	2.21<br>581	0	2.99<br>E-05
GO_CELL_CHEMOTAXIS	255	0.51<br>1257	2.21<br>2938	0	2.95<br>E-05
GO_REGULATION_OF_LYMPHOCYTE_DIFFERENTIATION	161	0.53<br>8073	2.21<br>0085	0	2.90<br>E-05
GO_REGULATION_OF_LEUKOCYTE_MIGRATION	182	0.52<br>652	2.20<br>9654	0	2.86<br>E-05
GO_NEGATIVE_REGULATION_OF_CELL_ACTIVATION	176	0.53<br>1529	2.20<br>7925	0	2.82<br>E-05
GO_REGULATION_OF_NEUTROPHIL_MIGRATION	36	0.66<br>1333	2.20<br>6015	0	4.17<br>E-05
GO_REGULATION_OF_T_CELL_DIFFERENTIATION	132	0.54<br>546	2.20<br>5676	0	4.11<br>E-05
GO_POSITIVE_REGULATION_OF_PHAGOCYTOSIS	62	0.60<br>7817	2.20<br>5594	0	4.05<br>E-05
GO_INTERLEUKIN_6_PRODUCTION	124	0.55<br>1472	2.20<br>3345	0	4.00<br>E-05
GO_NEGATIVE_REGULATION_OF_T_CELL_RECEPTOR_SIGNALING_PATHWAY	22	0.75<br>819	2.20<br>2546	0	3.95<br>E-05
GO_THYMIC_T_CELL_SELECTION	22	0.73<br>4441	2.19<br>8023	0	3.90<br>E-05
GO_POSITIVE_REGULATION_OF_TUMOR_NECROSIS_FACTOR_BIOSYNTHETIC_PROCESS	24	0.72<br>4791	2.19<br>4378	0	3.85<br>E-05
GO_MEMBRANE_INVAGINATION	61	0.61<br>1614	2.19<br>3723	0	3.80<br>E-05
GO_ALPHA_BETA_T_CELL_ACTIVATION	129	0.53<br>8771	2.18<br>8612	0	3.75<br>E-05
GO_REGULATION_OF_PHAGOCYTOSIS	85	0.57<br>288	2.18<br>8126	0	3.70<br>E-05
GO_T_CELL_DIFFERENTIATION	227	0.51<br>2756	2.18<br>5765	0	3.66<br>E-05
GO_LEUKOCYTE_DIFFERENTIATION	483	0.49<br>0662	2.18<br>3393	0	4.85<br>E-05
GO_B_CELL_MEDIATED_IMMUNITY	110	0.54<br>6517	2.18<br>2367	0	4.80<br>E-05
GO_REGULATION_OF_ALPHA_BETA_T_CELL_ACTIVATION	84	0.57<br>2117	2.18<br>2312	0	4.74<br>E-05
GO_REGULATION_OF_LEUKOCYTE_APOPTOTIC_PROCESS	76	0.58<br>2758	2.17<br>8112	0	4.68<br>E-05
GO_T_CELL_DIFFERENTIATION_INVOLVED_IN_IMMUNE_RESPONSE	64	0.59<br>1003	2.17<br>776	0	4.63<br>E-05

GO_MACROPHAGE_ACTIVATION	87	0.56 4028	2.17 5658	0	4.58 E-05
GO_POSITIVE_REGULATION_OF_HEMOPOIESIS	174	0.51 8853	2.17 1401	0	4.53 E-05
GO_POSITIVE_REGULATION_OF_CYTOKINE_BIOSYNTHETIC_PROCESS	36	0.65 1235	2.17 0418	0	4.48 E-05
GO_T_CELL_LINEAGE_COMMITMENT	26	0.69 9669	2.16 6321	0	5.54 E-05
GO_ADAPTIVE_IMMUNE_RESPONSE_BASED_ON_SOMATIC_RECOMBINATION_OF_IMMUNE_RECEPTORS_ BUILT_FROM_IMMUNOGLOBULIN_SUPERFAMILY_DOMAINS	241	0.50 6708	2.16 525	0	5.48 E-05
GO_ANTIGEN_RECEPTOR_MEDIATED_SIGNALING_PATHWAY	223	0.50 7932	2.16 5066	0	5.42 E-05
GO_POSITIVE_REGULATION_OF_INTERFERON_GAMMA_PRODUCTION	61	0.59 4526	2.16 4817	0	5.36 E-05
GO_SUPEROXIDE_ANION_GENERATION	34	0.66 37	2.16 4041	0	5.31 E-05
GO_MONONUCLEAR_CELL_MIGRATION	79	0.57 0289	2.15 217	0	5.25 E-05
GO_ACTIVATION_OF_IMMUNE_RESPONSE	400	0.48 5214	2.14 9173	0	5.20 E-05
GO_LEUKOCYTE_MIGRATION_INVOLVED_IN_INFLAMMATORY_RESPONSE	15	0.78 2733	2.14 8112	0	5.14 E-05
GO_IMMUNE_RESPONSE_REGULATING_SIGNALING_PATHWAY	356	0.48 916	2.14 5424	0	6.11 E-05
GO_LEUKOCYTE_APOPTOTIC_PROCESS	97	0.55 4626	2.14 5368	0	6.05 E-05
GO_CD4_POSITIVE_ALPHA_BETA_T_CELL_DIFFERENTIATION	70	0.57 5858	2.14 3602	0	5.99 E-05
GO_MYELOID_DENDRITIC_CELL_ACTIVATION	26	0.69 9618	2.14 1265	0	5.93 E-05
GO_REGULATION_OF_LYMPHOCYTE_APOPTOTIC_PROCESS	51	0.60 9797	2.13 8035	0	6.86 E-05
GO_NEGATIVE_REGULATION_OF_TUMOR_NECROSIS_FACTOR_SUPERFAMILY_CYTOKINE_PRODUCTION	56	0.59 7583	2.13 5595	0	7.76 E-05
GO_ALPHA_BETA_T_CELL_DIFFERENTIATION	95	0.55 0483	2.13 2121	0	7.69 E-05
GO_RESPONSE_TO_CHEMOKINE	84	0.55 57	2.13 1028	0	8.57 E-05
GO_INTERFERON_GAMMA_MEDIATED_SIGNALING_PATHWAY	84	0.55 9119	2.13 0851	0	8.48 E-05
GO_TUMOR_NECROSIS_FACTOR_SUPERFAMILY_CYTOKINE_PRODUCTION	149	0.51 8556	2.12 7194	0	8.41 E-05
GO_POSITIVE_REGULATION_OF_INTERLEUKIN_10_PRODUCTION	32	0.65 7525	2.11 9292	0	9.27 E-05

GO_ALPHA_BETA_T_CELL_ACTIVATION_INVOLVED_IN_IMMUNE_RESPONSE	57	0.58 6809	2.11 8717	0	9.19 E-05
GO_APOPTOTIC_CELL_CLEARANCE	44	0.62 3039	2.11 4624	0	9.10 E-05
GO_ANTIGEN_PROCESSING_AND_PRESENTATION_OF_PEPTIDE_OR_POLYSACCHARIDE_ANTIGEN_VIA_M HC_CLASS_II	92	0.54 9479	2.11 1593	0	1.08 E-04
GO_REGULATION_OF_GRANULOCYTE_CHEMOTAXIS	41	0.62 5649	2.10 0096	0	1.52 E-04
GO_REGULATION_OF_LEUKOCYTE_MEDIATED_IMMUNITY	186	0.50 1449	2.09 4599	0	1.94 E-04
GO_NATURAL_KILLER_CELL_DIFFERENTIATION	21	0.71 9445	2.09 3546	0	1.93 E-04
GO_CELLULAR_EXTRAVASATION	64	0.56 7777	2.09 2932	0	2.00 E-04
GO_POSITIVE_REGULATION_OF_INTERLEUKIN_6_PRODUCTION	76	0.55 1867	2.09 1817	0	2.07 E-04
GO_INTEGRIN_MEDIATED_SIGNALING_PATHWAY	98	0.53 0638	2.09 176	0	2.05 E-04
GO_LEUKOCYTE_MEDIATED_CYTOTOXICITY	96	0.53 5166	2.08 7774	0	2.12 E-04
GO_REGULATION_OF_LYMPHOCYTE_CHEMOTAXIS	25	0.68 3938	2.08 4562	0	2.18 E-04
GO_NEGATIVE_REGULATION_OF_IMMUNE_SYSTEM_PROCESS	382	0.47 3154	2.08 1801	0	2.25 E-04
GO_CYTOKINE_SECRETION	103	0.53 6491	2.07 97	0	2.31 E-04
GO_REGULATION_OF_B_CELL_ACTIVATION	109	0.52 1724	2.07 8072	0	2.29 E-04
GO_ALPHA_BETA_T_CELL_PROLIFERATION	31	0.65 4139	2.07 5075	0	2.36 E-04
GO_REGULATION_OF_NEUTROPHIL_CHEMOTAXIS	27	0.66 8834	2.07 3943	0	2.42 E-04
GO_NEGATIVE_REGULATION_OF_CELL_CELL_ADHESION	155	0.49 7316	2.07 0665	0	2.56 E-04
GO_RESPONSE_TO_MOLECULE_OF_BACTERIAL_ORIGIN	295	0.47 815	2.06 6321	0	2.78 E-04
GO_NEGATIVE_REGULATION_OF_LEUKOCYTE_APOPTOTIC_PROCESS	43	0.60 7736	2.06 6242	0	2.76 E-04
GO_REGULATION_OF_MONOCYTE_DIFFERENTIATION	18	0.72 4955	2.06 5359	0	2.73 E-04
GO_INTERLEUKIN_8_PRODUCTION	72	0.55 3318	2.06 4677	0	2.71 E-04
GO_B_CELL_ACTIVATION	227	0.48 8913	2.06 3804	0	2.77 E-04

GO_NEUROINFLAMMATORY_RESPONSE	62	0.56<br>7238	2.06<br>1835	0	2.90<br>E-04
GO_FC_RECEPTOR_MEDIATED_STIMULATORY_SIGNALING_PATHWAY	80	0.54<br>5332	2.06<br>1776	0	2.88<br>E-04
GO_REGULATION_OF_MYELOID_LEUKOCYTE_MEDIATED_IMMUNITY	53	0.58<br>0476	2.05<br>5048	0	3.08<br>E-04
GO_LYMPHOCYTE_APOPTOTIC_PROCESS	68	0.55<br>7205	2.05<br>4822	0	3.06<br>E-04
GO_LEUKOCYTE_ACTIVATION_INVOLVED_IN_INFLAMMATORY_RESPONSE	42	0.60<br>3973	2.05<br>1811	0	3.41<br>E-04
GO_POSITIVE_REGULATION_OF_ALPHA_BETA_T_CELL_ACTIVATION	56	0.56<br>731	2.04<br>9973	0	3.60<br>E-04
GO_POSITIVE_REGULATION_OF_INTERLEUKIN_8_PRODUCTION	49	0.58<br>3878	2.04<br>5623	0	3.72<br>E-04
GO_INTERLEUKIN_12_PRODUCTION	51	0.57<br>6115	2.04<br>2001	0	4.20<br>E-04
GO_INTERLEUKIN_1_BETA_PRODUCTION	78	0.54<br>0712	2.03<br>4562	0	4.75<br>E-04
GO_INTERLEUKIN_2_PRODUCTION	48	0.58<br>1805	2.03<br>3078	0	4.71<br>E-04
GO_REGULATION_OF_LEUKOCYTE_DEGRANULATION	44	0.59<br>4485	2.03<br>2272	0.00<br>1279	4.82<br>E-04
GO_CD4_POSITIVE_OR_CD8_POSITIVE_ALPHA_BETA_T_CELL_LINEAGE_COMMITMENT	20	0.70<br>9071	2.03<br>2206	0	4.79<br>E-04
GO_TYPE_2_IMMUNE_RESPONSE	36	0.61<br>3294	2.03<br>1384	0	4.76<br>E-04
GO_POSITIVE_REGULATION_OF_TUMOR_NECROSIS_FACTOR_SUPERFAMILY_CYTOKINE_PRODUCTION	85	0.53<br>4326	2.03<br>0892	0	4.72<br>E-04
GO_CD8_POSITIVE_ALPHA_BETA_T_CELL_ACTIVATION	25	0.66<br>3707	2.02<br>7085	0	5.04<br>E-04
GO_POSITIVE_REGULATION_OF_LYMPHOCYTE_CHEMOTAXIS	21	0.69<br>4718	2.02<br>5358	0	5.27<br>E-04
GO_EOSINOPHIL_CHEMOTAXIS	18	0.72<br>1812	2.02<br>4064	0	5.37<br>E-04
GO_POSITIVE_REGULATION_OF_MACROPHAGE_MIGRATION	21	0.69<br>4793	2.02<br>3289	0	5.47<br>E-04
GO_POSITIVE_REGULATION_OF_INTERLEUKIN_4_PRODUCTION	23	0.69<br>1672	2.02<br>0692	0	5.64<br>E-04
GO_T_CELL_CHEMOTAXIS	25	0.66<br>7661	2.01<br>7918	0	5.73<br>E-04
GO_LEUKOCYTE_HOMEOSTASIS	78	0.53<br>6225	2.01<br>5926	0	5.76<br>E-04
GO_CELLULAR_RESPONSE_TO_MOLECULE_OF_BACTERIAL_ORIGIN	179	0.48<br>2988	2.01<br>4738	0	5.72<br>E-04

GO_DETECTION_OF_OTHER_ORGANISM	18	0.723448	2.014725	0	5.69E-04
GO_POSITIVE_REGULATION_OF_INTERLEUKIN_1_PRODUCTION	51	0.57339	2.013799	0	5.84E-04
GO_DETECTION_OF_BIOTIC_STIMULUS	34	0.611689	2.012907	0	5.87E-04
GO_CD4_POSITIVE_ALPHA_BETA_T_CELL_ACTIVATION	84	0.524208	2.009333	0	6.22E-04
GO_DETECTION_OF_EXTERNAL_BIOTIC_STIMULUS	23	0.686455	2.008252	0	6.24E-04
GO_MYELOID_LEUKOCYTE_DIFFERENTIATION	191	0.475993	2.008134	0	6.21E-04
GO_B_CELL_DIFFERENTIATION	125	0.502273	2.007046	0	6.36E-04
GO_POSITIVE_REGULATION_OF_CYTOKINE_PRODUCTION	403	0.455171	2.006411	0	6.38E-04
GO_GLIAL_CELL_ACTIVATION	50	0.577683	2.005353	0	6.40E-04
GO_ACTIVATED_T_CELL_PROLIFERATION	42	0.601511	2.004459	0	6.36E-04
GO_NEGATIVE_REGULATION_OF_INTERFERON_GAMMA_PRODUCTION	30	0.635515	2.003824	0	6.45E-04
GO_CHEMOKINE_PRODUCTION	81	0.521655	2.003218	0	6.47E-04
GO_REGULATION_OF_TYPE_2_IMMUNE_RESPONSE	28	0.63691	2.001842	0	6.55E-04
GO_T_CELL_RECEPTOR_SIGNALING_PATHWAY	183	0.481442	2.001219	0	6.57E-04
GO_REGULATION_OF_CELL_KILLING	84	0.52295	2.000648	0	6.71E-04
GO_NEGATIVE_REGULATION_OF_ALPHA_BETA_T_CELL_ACTIVATION	32	0.620432	1.998819	0	7.21E-04
GO_INTERLEUKIN_1_PRODUCTION	89	0.52375	1.994079	0	8.05E-04
GO_NEGATIVE_REGULATION_OF_INTERLEUKIN_6_PRODUCTION	36	0.608016	1.992638	0	8.36E-04
GO_HETEROTYPIC_CELL_CELL_ADHESION	60	0.550619	1.99257	0	8.37E-04
GO_POSITIVE_REGULATION_OF_ALPHA_BETA_T_CELL_PROLIFERATION	20	0.693478	1.990034	0	8.61E-04
GO_NATURAL_KILLER_CELL_ACTIVATION	69	0.532914	1.986396	0	9.61E-04
GO_IMMUNOGLOBULIN_PRODUCTION_INVOLVED_IN_IMMUNOGLOBULIN_MEDIATED_IMMUNE_RESPONSE	51	0.566207	1.984737	0	0.001001

GO_ANTIGEN_PROCESSING_AND_PRESENTATION	213	0.47 2178	1.98 1726	0	0.00 1036
GO_ACUTE_INFLAMMATORY_RESPONSE_TO_ANTIGENIC_STIMULUS	21	0.68 6965	1.98 1096	0	0.00 1047
GO_REGULATION_OF_ALPHA_BETA_T_CELL_DIFFERENTIATION	57	0.55 5585	1.97 8011	0	0.00 1086
GO_CELLULAR_DEFENSE_RESPONSE	50	0.56 1508	1.97 7182	0	0.00 1097
GO_POSITIVE_REGULATION_OF_INFLAMMATORY_RESPONSE	134	0.48 8395	1.97 5596	0	0.00 1125
GO_LYSOSOME_LOCALIZATION	65	0.53 5488	1.97 5575	0	0.00 1118
GO_POSITIVE_REGULATION_OF_IMMUNE_EFFECTOR_PROCESS	192	0.46 795	1.96 8357	0	0.00 1245
GO_POSITIVE_REGULATION_OF_ALPHA_BETA_T_CELL_DIFFERENTIATION	41	0.57 8897	1.96 722	0	0.00 1266
GO_MAST_CELL_MEDIATED_IMMUNITY	46	0.56 6286	1.96 6315	0	0.00 1291
GO_INTERLEUKIN_13_PRODUCTION	20	0.68 1554	1.96 4891	0	0.00 1317
GO_REGULATION_OF_INFLAMMATORY_RESPONSE	324	0.44 9449	1.96 1084	0	0.00 1402
GO_REGULATION_OF_HEMOPOIESIS	403	0.44 5387	1.95 9958	0	0.00 141
GO_REGULATION_OF_LYMPHOCYTE_MEDIATED_IMMUNITY	136	0.48 609	1.95 942	0	0.00 1403
GO_REGULATION_OF_SUPEROXIDE_ANION_GENERATION	21	0.66 3634	1.95 4501	0	0.00 1507
GO_REGULATION_OF_B_CELL_MEDIATED_IMMUNITY	46	0.57 0415	1.95 3829	0	0.00 1526
GO_NEGATIVE_REGULATION_OF_PEPTIDYL_TYROSINE_PHOSPHORYLATION	45	0.57 4058	1.95 3325	0	0.00 1523
GO_B_CELL_ACTIVATION_INVOLVED_IN_IMMUNE_RESPONSE	69	0.52 6694	1.95 0828	0	0.00 1589
GO_REGULATION_OF_MYELOID_LEUKOCYTE_DIFFERENTIATION	109	0.49 5192	1.94 9691	0	0.00 1596
GO_NEGATIVE_REGULATION_OF_IMMUNE_RESPONSE	142	0.47 9957	1.94 7314	0	0.00 1629
GO_DENDRITIC_CELL_MIGRATION	23	0.65 8673	1.94 7142	0	0.00 1621
GO_REGULATION_OF_IMMUNE_EFFECTOR_PROCESS	357	0.43 9426	1.94 6235	0	0.00 1633
GO_CELL_JUNCTION_DISASSEMBLY	19	0.68 7291	1.94 3913	0	0.00 1671

GO_NEGATIVE_REGULATION_OF_CYTOKINE_PRODUCTION	232	0.45643	1.943649	0	0.001667
GO_ANTIGEN_PROCESSING_AND_PRESENTATION_OF_PEPTIDE_ANTIGEN	175	0.463767	1.942159	0	0.001699
GO_REGULATION_OF_CD8_POSITIVE_ALPHA_BETA_T_CELL_ACTIVATION	17	0.695697	1.940646	0	0.001736
GO_POSITIVE_REGULATION_OF_B_CELL_MEDIATED_IMMUNITY	31	0.605321	1.940349	0	0.001733
GO_NEGATIVE_REGULATION_OF_INTERLEUKIN_10_PRODUCTION	16	0.714511	1.940082	0.001473	0.001739
GO_REGULATION_OF_LEUKOCYTE_MEDIATED_CYTOTOXICITY	69	0.520618	1.939245	0	0.001745
GO_CELLULAR_RESPONSE_TO_BIOTIC_STIMULUS	200	0.456671	1.936701	0	0.001771
GO_NATURAL_KILLER_CELL_MEDIATED_IMMUNITY	57	0.539032	1.936369	0	0.001777
GO_PINOCYTOSIS	20	0.662529	1.934713	0	0.001822
GO_NEGATIVE_REGULATION_OF_REGULATED_SECRETORY_PATHWAY	22	0.655457	1.933004	0	0.001872
GO_REGULATION_OF_MACROPHAGE_MIGRATION	35	0.589817	1.932434	0	0.001873
GO_T_CELL_MEDIATED_IMMUNITY	98	0.500803	1.930382	0	0.001975
GO_POSITIVE_REGULATION_OF_LYMPHOCYTE_APOPTOTIC_PROCESS	15	0.72016	1.929704	0	0.001984
GO_POSITIVE_REGULATION_OF_TYPE_2_IMMUNE_RESPONSE	15	0.716932	1.927717	0	0.002008
GO_IMMUNOGLOBULIN_SECRETION	19	0.673252	1.927637	0.001439	0.001999
GO_NEGATIVE_REGULATION_OF_CELL_ADHESION	245	0.452838	1.924303	0	0.002117
GO_POSITIVE_REGULATION_OF_CALCIUM_MEDIATED_SIGNALING	39	0.577685	1.924199	0	0.002107
GO_NEGATIVE_REGULATION_OF_EXOCYTOSIS	30	0.611381	1.921329	0	0.002215
GO_CYTOKINE_PRODUCTION_INVOLVED_IN_INFLAMMATORY_RESPONSE	45	0.556727	1.920053	0	0.002265
GO_T_CELL_HOMEOSTASIS	36	0.589736	1.919012	0	0.002287
GO_CELL_KILLING	148	0.466573	1.915337	0	0.002407
GO_REGULATION_OF_ADAPTIVE_IMMUNE_RESPONSE	151	0.466833	1.9144	0	0.002437

GO_MYELOID_CELL_DIFFERENTIATION	341	0.437918	1.913119	0	0.002462
GO_ACTIN_POLYMERIZATION_OR_DEPOLYMERIZATION	179	0.458553	1.912687	0	0.002465
GO_T_HELPER_1_TYPE_IMMUNE_RESPONSE	41	0.574467	1.911594	0	0.00249
GO_T_CELL_DIFFERENTIATION_IN_THYMUS	71	0.513023	1.909394	0	0.002587
GO_CHRONIC_INFLAMMATORY_RESPONSE	19	0.654454	1.906452	0.002865	0.002729
GO_REGULATION_OF_B_CELL_DIFFERENTIATION	27	0.61065	1.904767	0	0.002765
GO_REGULATION_OF_B_CELL_RECEPTOR_SIGNALING_PATHWAY	22	0.652165	1.903639	0	0.002807
GO_MACROPHAGE_MIGRATION	47	0.540541	1.899017	0.001294	0.002958
GO_ORGANELLE_MEMBRANE_FUSION	87	0.496832	1.897605	0	0.003016
GO_POSITIVE_REGULATION_OF_GRANULOCYTE_CHEMOTAXIS	25	0.614542	1.891776	0	0.003324
GO_MEMBRANE_RAFT_ORGANIZATION	22	0.64073	1.889324	0	0.003437
GO_POSITIVE_REGULATION_OF_RESPONSE_TO_CYTOKINE_STIMULUS	47	0.54388	1.88932	0	0.003422
GO_REGULATION_OF_T_CELL_CYTOKINE_PRODUCTION	31	0.582745	1.884203	0	0.003706
GO_REGULATION_OF_HUMORAL_IMMUNE_RESPONSE	66	0.514667	1.882603	0	0.003742
GO_HUMORAL_IMMUNE_RESPONSE_MEDIATED_BY_CIRCULATING_IMMUNOGLOBULIN	46	0.538832	1.882405	0	0.003731
GO_POSITIVE_REGULATION_OF_ADAPTIVE_IMMUNE_RESPONSE	96	0.486505	1.879925	0	0.003877
GO_POSITIVE_REGULATION_OF_LEUKOCYTE_MEDIATED_IMMUNITY	118	0.465705	1.879427	0	0.003878
GO_POSITIVE_REGULATION_OF_NEUTROPHIL_MIGRATION	27	0.621079	1.877181	0	0.004002
GO_POSITIVE_REGULATION_OF_CYTOKINE_SECRETION	57	0.528549	1.876747	0	0.004002
GO_MACROPHAGE_DIFFERENTIATION	43	0.557597	1.875058	0.001277	0.004082
GO_POSITIVE_REGULATION_OF_CHEMOTAXIS	125	0.465204	1.873544	0	0.004162
GO_REGULATION_OF_CD4_POSITIVE_ALPHA_BETA_T_CELL_DIFFERENTIATION	43	0.542307	1.871898	0	0.004261

GO_LYMPHOID_PROGENITOR_CELL_DIFFERENTIATION	20	0.64 7802	1.87 0933	0	0.00 4298
GO_POSITIVE_REGULATION_OF_MYELOID_LEUKOCYTE_DIFFERENTIATION	52	0.53 1853	1.86 9906	0	0.00 4347
GO_REGULATION_OF_ISOTYPE_SWITCHING	29	0.58 7965	1.86 9548	0	0.00 4353
GO_CELL_SUBSTRATE_ADHESION	318	0.43 0243	1.86 6694	0	0.00 4561
GO_VASCULAR_ENDOTHELIAL_GROWTH_FACTOR_RECEPTOR_SIGNALING_PATHWAY	85	0.48 9572	1.86 5159	0	0.00 4641
GO_TOLERANCE_INDUCTION	21	0.64 5559	1.86 4771	0	0.00 4647
GO_REGULATION_OF_MAST_CELL_ACTIVATION_INVOLVED_IN_IMMUNE_RESPONSE	31	0.58 2984	1.86 1766	0	0.00 4831
GO_ASTROCYTE_DEVELOPMENT	40	0.55 1011	1.86 117	0.00 1348	0.00 4884
GO_POSITIVE_REGULATION_OF_CD4_POSITIVE_ALPHA_BETA_T_CELL_DIFFERENTIATION	26	0.61 4167	1.85 9909	0.00 1418	0.00 4953
GO_REGULATION_OF_CD4_POSITIVE_ALPHA_BETA_T_CELL_ACTIVATION	53	0.52 4617	1.85 9638	0	0.00 4946
GO_POSITIVE_REGULATION_OF_ANTIGEN_RECEPTOR_MEDIATED_SIGNALING_PATHWAY	18	0.66 5807	1.85 9279	0	0.00 4934
GO_NEGATIVE_REGULATION_OF_CYTOKINE_PRODUCTION_INVOLVED_IN_IMMUNE_RESPONSE	24	0.62 0003	1.85 9048	0	0.00 4934
GO_B_CELL_PROLIFERATION	86	0.48 9763	1.85 7516	0	0.00 5026
GO_REGULATION_OF_CYTOKINE_PRODUCTION_INVOLVED_IN_IMMUNE_RESPONSE	76	0.49 3886	1.85 7345	0	0.00 5018
GO_REGULATION_OF_CALCIUM_MEDIATED_SIGNALING	84	0.48 7257	1.85 5789	0	0.00 5124
GO_POSITIVE_REGULATION_OF_CD4_POSITIVE_ALPHA_BETA_T_CELL_ACTIVATION	32	0.57 7463	1.85 5202	0	0.00 5139
GO_MYELOID_DENDRITIC_CELL_DIFFERENTIATION	16	0.67 2624	1.85 1922	0.00 1443	0.00 54
GO_BASEMENT_MEMBRANE_ORGANIZATION	28	0.59 4438	1.85 059	0	0.00 5508
GO_MONONUCLEAR_CELL_DIFFERENTIATION	33	0.57 0431	1.85 008	0	0.00 5522
GO_T_HELPER_CELL_LINEAGE_COMMITMENT	15	0.69 0078	1.84 9726	0	0.00 5543
GO_REGULATION_OF_CELL_SHAPE	134	0.45 1516	1.84 7096	0	0.00 5752
GO_REGULATION_OF_INFLAMMATORY_RESPONSE_TO_ANTIGENIC_STIMULUS	26	0.60 0347	1.84 7015	0.00 141	0.00 573

GO_LEUKOCYTE_ADHESION_TO_VASCULAR_ENDOTHELIAL_CELL	41	0.539541	1.846481	0.001312	0.005739
GO_ORGANELLE_FUSION	111	0.467681	1.84571	0	0.005744
GO_REGULATION_OF_B_CELL_APOPTOTIC_PROCESS	19	0.639711	1.844798	0.001437	0.005787
GO_NEGATIVE_REGULATION_OF_LEUKOCYTE_MEDIATED_IMMUNITY	51	0.526968	1.843141	0	0.005867
GO_MAST_CELL_ACTIVATION	57	0.5178	1.843022	0	0.005852
GO_B_CELL_APOPTOTIC_PROCESS	25	0.60227	1.84167	0	0.005943
GO_NEGATIVE_REGULATION_OF_LEUKOCYTE_DIFFERENTIATION	91	0.478315	1.840791	0	0.005988
GO_POSITIVE_REGULATION_OF_ERK1_AND_ERK2_CASCADE	175	0.445226	1.839779	0	0.006063
GO_ACUTE_INFLAMMATORY_RESPONSE	101	0.468837	1.839559	0	0.006059
GO_POSITIVE_REGULATION_OF_LYMPHOCYTE_MEDIATED_IMMUNITY	92	0.472742	1.834572	0	0.006432
GO_INTERLEUKIN_4_PRODUCTION	32	0.578623	1.834107	0	0.006449
GO_LYTIC_VACUOLE_ORGANIZATION	55	0.511185	1.833734	0	0.006447
GO_MEMBRANE_FUSION	133	0.454533	1.831246	0	0.006708
GO_REGULATION_OF_CELL_SUBSTRATE_ADHESION	190	0.433435	1.828466	0	0.006953
GO_COMPLEMENT_ACTIVATION	64	0.494506	1.828443	0	0.006928
GO_REGULATION_OF_CHEMOTAXIS	190	0.436561	1.826906	0	0.007026
GO_ENDOSOME_TO_LYSOSOME_TRANSPORT	41	0.540898	1.824952	0	0.007209
GO_POSITIVE_REGULATION_OF_B_CELL_ACTIVATION	69	0.496027	1.82492	0	0.007187
GO_POSITIVE_REGULATION_OF_ACUTE_INFLAMMATORY_RESPONSE	27	0.585107	1.824214	0.001401	0.007226
GO_T_CELL_APOPTOTIC_PROCESS	45	0.530189	1.823296	0	0.007282
GO_NEGATIVE_REGULATION_OF_T_CELL_DIFFERENTIATION	37	0.553988	1.821816	0	0.007399
GO_LYMPHOCYTE_HOMEOSTASIS	58	0.511743	1.820961	0	0.007443

GO_REGULATION_OF_CELL_MATRIX_ADHESION	109	0.457671	1.815534	0	0.008015
GO_REGULATION_OF_ACTIN_FILAMENT_LENGTH	153	0.438857	1.81469	0	0.008061
GO_ERK1_AND_ERK2_CASCADE	268	0.420259	1.813919	0	0.008124
GO_CYTOKINE_PRODUCTION_INVOLVED_IN_IMMUNE_RESPONSE	95	0.461756	1.813413	0	0.008148
GO_REGULATION_OF_ACTIN_CYTOSKELETON_REORGANIZATION	37	0.552148	1.812873	0.001406	0.008189
GO_REGULATION_OF_MONONUCLEAR_CELL_MIGRATION	41	0.527225	1.812859	0	0.008161
GO_PHAGOSOME_MATURATION	45	0.530268	1.812549	0	0.008157
GO_DENDRITIC_CELL_CHEMOTAXIS	18	0.635237	1.810563	0	0.008329
GO_ENTRY_INTO_HOST	130	0.448075	1.809737	0	0.008389
GO_NEGATIVE_REGULATION_OF_LYMPHOCYTE_DIFFERENTIATION	46	0.514913	1.809514	0.002571	0.008391
GO_REGULATION_OF_NATURAL_KILLER_CELL_ACTIVATION	30	0.574097	1.807814	0.001399	0.008543
GO_NEGATIVE_REGULATION_OF_INFLAMMATORY_RESPONSE	129	0.450687	1.806655	0	0.00868
GO_MOVEMENT_IN_HOST_ENVIRONMENT	150	0.445306	1.806012	0	0.008742
GO_REGULATION_OF_B_CELL_PROLIFERATION	59	0.497391	1.803401	0.001255	0.008982
GO_LIPOPOLYSACCHARIDE_MEDIATED_SIGNALING_PATHWAY	55	0.510753	1.802831	0	0.009026
GO_REGULATION_OF_ACUTE_INFLAMMATORY_RESPONSE	44	0.536408	1.801701	0.001287	0.009143
GO_POSITIVE_REGULATION_OF_CELL_KILLING	56	0.498745	1.798948	0	0.009506
GO_REGULATION_OF_MACROPHAGE_CHEMOTAXIS	23	0.592918	1.798615	0.001435	0.009528
GO_COAGULATION	303	0.414037	1.797461	0	0.009612
GO_NEGATIVE_REGULATION_OF_MACROPHAGE_ACTIVATION	16	0.66046	1.795803	0.004471	0.009805
GO_REGULATION_OF_MACROPHAGE_ACTIVATION	50	0.511502	1.794466	0.001305	0.009914
GO_REGULATION_OF_MAST_CELL_ACTIVATION	40	0.526021	1.793153	0.001318	0.010053

GO_PRODUCTION_OF_MOLECULAR_MEDIATOR_INVOLVED_IN_INFLAMMATORY_RESPONSE	67	0.484208	1.792621	0	0.010092
GO_POSITIVE_REGULATION_OF_MYELOID_CELL_DIFFERENTIATION	84	0.474947	1.791868	0	0.01017
GO_REGULATION_OF_SYSTEMIC_ARTERIAL_BLOOD_PRESSURE_BY_RENIN_ANGIOTENSIN	25	0.587102	1.791753	0.005764	0.010147
GO_MATURE_B_CELL_DIFFERENTIATION	26	0.581088	1.788912	0.001433	0.010461
GO_REGULATION_OF_MEMBRANE_PROTEIN_ECTODOMAIN_PROTEOLYSIS	21	0.615849	1.788479	0.00419	0.010502
GO_NEGATIVE_REGULATION_OF_IMMUNE_EFFECTOR_PROCESS	114	0.455754	1.787255	0	0.010629
GO_REGULATION_OF_PROTEIN_LOCALIZATION_TO_PLASMA_MEMBRANE	89	0.46006	1.785521	0	0.010854
GO_RESPONSE_TO_TUMOR_NECROSIS_FACTOR	270	0.415012	1.783573	0	0.011085
GO_REGULATION_OF_T_CELL_CHEMOTAXIS	17	0.651483	1.782435	0.001534	0.011218
GO_MYELOID_CELL_HOMEOSTASIS	129	0.441911	1.780089	0	0.011548
GO_POSITIVE_REGULATION_OF_INTERLEUKIN_2_PRODUCTION	25	0.58961	1.779895	0.00135	0.011547
GO_ACTIN_FILAMENT_DEPOLYMERIZATION	51	0.5046	1.779021	0	0.011624
GO_POSITIVE_REGULATION_OF_RESPONSE_TO_EXTERNAL_STIMULUS	450	0.403897	1.777505	0	0.011852
GO_REGULATION_OF_T_CELL_APOPTOTIC_PROCESS	30	0.557991	1.776953	0.001479	0.011893
GO_HUMORAL_IMMUNE_RESPONSE	223	0.419322	1.774268	0	0.012285
GO_INTERACTION_WITH_HOST	187	0.417768	1.772131	0	0.012552
GO_NEGATIVE_REGULATION_OF_CELLULAR_COMPONENT_MOVEMENT	278	0.408549	1.770914	0	0.012713
GO_REGULATION_OF_PEPTIDYL_TYROSINE_PHOSPHORYLATION	235	0.414282	1.770778	0	0.012701
GO_NEGATIVE_REGULATION_OF_HEMOPOIESIS	126	0.43737	1.770628	0	0.012681
GO_NEGATIVE_REGULATION_OF_MAPK_CASCADE	154	0.432882	1.769618	0	0.012824
GO_REGULATION_OF_T_CELL_MEDIATED_IMMUNITY	68	0.481108	1.768581	0	0.012957
GO_REGULATION_OF_PRODUCTION_OF_MOLECULAR_MEDIATOR_OF_IMMUNE_RESPONSE	124	0.444529	1.768255	0	0.012991

GO_SUBSTRATE_ADHESION_DEPENDENT_CELL_SPREADING	93	0.45<br>6121	1.76<br>547	0.00<br>2358	0.01<br>3364
GO_POSITIVE_REGULATION_OF_T_CELL_CYTOKINE_PRODUCTION	19	0.60<br>9047	1.76<br>3069	0.00<br>4405	0.01<br>377
GO_POSITIVE_REGULATION_OF_CHEMOKINE_PRODUCTION	55	0.49<br>5263	1.76<br>0298	0.00<br>1256	0.01<br>4184
GO_NEGATIVE_REGULATION_OF_ADAPTIVE_IMMUNE_RESPONSE	47	0.50<br>444	1.75<br>7414	0	0.01<br>4698
GO_PH_REDUCTION	53	0.49<br>7137	1.75<br>6732	0.00<br>1285	0.01<br>4765
GO_REGULATION_OF_SYNCYTIUM_FORMATION_BY_PLASMA_MEMBRANE_FUSION	26	0.57<br>5044	1.75<br>586	0.00<br>1362	0.01<br>4901
GO_NEGATIVE_REGULATION_OF_CD4_POSITIVE_ALPHA_BETA_T_CELL_ACTIVATION	24	0.58<br>3538	1.75<br>4122	0.00<br>4115	0.01<br>5258
GO_INTERLEUKIN_7_MEDIATED_SIGNALING_PATHWAY	17	0.63<br>284	1.75<br>2938	0.00<br>4666	0.01<br>5443
GO_ENGULFMENT_OF_APOPTOTIC_CELL	15	0.65<br>0715	1.75<br>0655	0.00<br>8772	0.01<br>5859
GO_REGULATION_OF_T_HELPER_1_TYPE_IMMUNE_RESPONSE	26	0.57<br>1877	1.74<br>9945	0.00<br>411	0.01<br>5957
GO_FC_RECEPTOR_SIGNALING_PATHWAY	169	0.41<br>7327	1.74<br>9322	0	0.01<br>6056
GO_ACTIN_CYTOSKELETON_REORGANIZATION	93	0.45<br>225	1.74<br>896	0	0.01<br>6077
GO_POSITIVE_REGULATION_OF_NF_KAPPAB_TRANSCRIPTION_FACTOR_ACTIVITY	145	0.42<br>5236	1.74<br>878	0.00<br>1125	0.01<br>61
GO_REGULATION_OF_IMMUNOGLOBULIN_PRODUCTION	54	0.49<br>8856	1.74<br>8402	0	0.01<br>613
GO_INOSITOL_PHOSPHATE_BIOSYNTHETIC_PROCESS	21	0.60<br>2027	1.74<br>802	0.00<br>554	0.01<br>6162
GO_POSITIVE_REGULATION_OF_PEPTIDE_SECRETION	196	0.41<br>6818	1.74<br>6387	0	0.01<br>6407
GO_GLIAL_CELL_APOPTOTIC_PROCESS	15	0.64<br>8628	1.74<br>5926	0.00<br>4484	0.01<br>6461
GO_REGULATION_OF_INNATE_IMMUNE_RESPONSE	267	0.40<br>2754	1.74<br>5505	0	0.01<br>6475
GO_POSITIVE_REGULATION_OF_LEUKOCYTE_ADHESION_TO_VASCULAR_ENDOTHELIAL_CELL	19	0.61<br>3552	1.74<br>5066	0.00<br>1447	0.01<br>6497
GO_NEGATIVE_REGULATION_OF_DEFENSE_RESPONSE	194	0.41<br>6694	1.74<br>4975	0	0.01<br>6464
GO_POSITIVE_REGULATION_OF_EXTRINSIC_APOPTOTIC_SIGNALING_PATHWAY	50	0.49<br>5673	1.74<br>4507	0.00<br>1271	0.01<br>6503
GO_NEGATIVE_REGULATION_OF_LEUKOCYTE_MIGRATION	39	0.51<br>7401	1.74<br>4272	0.00<br>2649	0.01<br>6493

GO_MODIFICATION_OF_SYNAPTIC_STRUCTURE	23	0.594902	1.743316	0.008403	0.016647
GO_CHEMOKINE_BIOSYNTHETIC_PROCESS	15	0.653308	1.742113	0.001495	0.016845
GO_PLATELET_ACTIVATION	146	0.427054	1.740581	0	0.017101
GO_REGULATION_OF_ACTIN_FILAMENT_ORGANIZATION	225	0.410841	1.739096	0	0.017388
GO_RELEASE_OF_CYTOCHROME_C_FROM_MITOCHONDRIA	53	0.488179	1.738357	0.001305	0.017483
GO_NEGATIVE_REGULATION_OF_LEUKOCYTE_MEDIATED_CYTOTOXICITY	20	0.603339	1.738092	0.003021	0.017513
GO_REGULATION_OF_MYELOID_CELL_DIFFERENTIATION	193	0.413069	1.737771	0	0.017554
GO_RECEPTOR_SIGNALING_PATHWAY_VIA_STAT	144	0.42497	1.732489	0	0.018579
GO_MACROPHAGE_CHEMOTAXIS	34	0.532818	1.728947	0.003922	0.019367
GO_POSITIVE_REGULATION_OF_JNK_CASCADE	126	0.425184	1.72758	0	0.019686
GO_REGULATION_OF_GTPASE_ACTIVITY	376	0.392732	1.726301	0	0.019932
GO_REGULATION_OF_ACTIN_FILAMENT_BASED_PROCESS	336	0.395743	1.726184	0	0.019899
GO_REGULATION_OF_CELLULAR_EXTRAVASATION	31	0.543783	1.726056	0.002755	0.019878
GO_T_CELL_CYTOKINE_PRODUCTION	42	0.508448	1.724904	0.001295	0.02008
GO_NEGATIVE_REGULATION_OF_PRODUCTION_OF_MOLECULAR_MEDIATOR_OF_IMMUNE_RESPONSE	35	0.538066	1.723849	0.00267	0.020286
GO_REGULATION_OF_EXTRACELLULAR_MATRIX_ORGANIZATION	34	0.524312	1.723463	0.004027	0.020319
GO_POSITIVE_REGULATION_OF_CALCIUM_ION_IMPORT	19	0.606015	1.721642	0.008889	0.020805
GO_POSITIVE_REGULATION_OF_GLIOGENESIS	65	0.479599	1.717735	0.001284	0.021608
GO_CD40_SIGNALING_PATHWAY	15	0.646448	1.717725	0.003115	0.02155
GO_GRANULOCYTE_DIFFERENTIATION	34	0.529609	1.716487	0.001366	0.021757
GO_TISSUE_REMODELING	152	0.42084	1.716466	0	0.021701
GO_NEGATIVE_REGULATION_OF_ENDOCYTOSIS	39	0.510216	1.71485	0.00406	0.02205

GO_WOUND_HEALING	461	0.386492	1.712691	0	0.022576
GO_POSITIVE_REGULATION_OF_DEFENSE_RESPONSE	328	0.39241	1.710635	0	0.023018
GO_REGULATION_OF_T_HELPER_CELL_DIFFERENTIATION	33	0.525842	1.710506	0.001364	0.023002
GO_DEFENSE_RESPONSE_TO_BACTERIUM	200	0.40689	1.710415	0	0.022946
GO_TUMOR_NECROSIS_FACTOR_MEDIATED_SIGNALING_PATHWAY	153	0.413463	1.707491	0	0.0236
GO_REGULATION_OF_AMINO_ACID_TRANSPORT	35	0.517422	1.705603	0.006579	0.024116
GO_MYELOID_CELL_DEVELOPMENT	58	0.475433	1.704868	0	0.024262
GO_POSITIVE_REGULATION_OF_NATURAL_KILLER_CELL_ACTIVATION	19	0.604471	1.704523	0.012552	0.024302
GO_REGULATION_OF_CELL_SUBSTRATE_JUNCTION_ORGANIZATION	59	0.470557	1.704039	0.002591	0.024368
GO_PROGRAMMED_NECROTIC_CELL_DEATH	39	0.499433	1.703535	0.001314	0.024435
GO_POSITIVE_REGULATION_OF_ESTABLISHMENT_OF_PROTEIN_LOCALIZATION	340	0.387422	1.703477	0	0.024393
GO_RESPONSE_TO_INTERLEUKIN_1	189	0.406178	1.701985	0	0.024779
GO_NEGATIVE_REGULATION_OF_MAP_KINASE_ACTIVITY	72	0.460444	1.701872	0.001221	0.024752
GO_T_HELPER_2_CELL_CYTOKINE_PRODUCTION	15	0.63431	1.701687	0.010417	0.024734
GO_POSITIVE_REGULATION_OF_RELEASE_OF_SEQUESTERED_CALCIUM_ION_INTO_CYTOSOL	36	0.520234	1.701347	0.002567	0.024749
GO_REGULATION_OF_ERBB_SIGNALING_PATHWAY	84	0.44133	1.701297	0.001203	0.024693
GO_NEGATIVE_REGULATION_OF_INNATE_IMMUNE_RESPONSE	55	0.472336	1.700721	0	0.024784
GO_PRODUCTION_OF_MOLECULAR_MEDIATOR_OF_IMMUNE_RESPONSE	187	0.407807	1.700374	0	0.0248
GO_NEGATIVE_REGULATION_OF_B_CELL_PROLIFERATION	17	0.609459	1.700045	0.011799	0.024819
GO_CELL_MATRIX_ADHESION	203	0.404357	1.699997	0	0.024774
GO_ENDOCYTIC_RECYCLING	42	0.499227	1.698901	0.003932	0.025026
GO_REGULATION_OF_OSTEOCLAST_DIFFERENTIATION	60	0.475158	1.698752	0.001253	0.025008

GO_POSITIVE_REGULATION_OF_PROTEIN_KINASE_B_SIGNALING	159	0.414251	1.698388	0	0.025037
GO_IMMUNOGLOBULIN_PRODUCTION	100	0.436857	1.696441	0	0.025582
GO_VACUOLE_ORGANIZATION	133	0.420009	1.696288	0	0.025553
GO_REGULATION_OF_CALCIUM_ION_TRANSPORT_INTO_CYTOSOL	87	0.441746	1.696049	0.00346	0.025562
GO_NEGATIVE_REGULATION_OF_INTERLEUKIN_2_PRODUCTION	19	0.593284	1.695563	0.017467	0.025629
GO_REGULATION_OF_VESICLE_FUSION	27	0.550435	1.695038	0.001422	0.025691
GO_ACTIVATION_OF_CYSTEINE_TYPE_ENDOPEPTIDASE_ACTIVITY_INVOLVED_IN_APOPTOTIC_PROCESS	74	0.452638	1.693568	0	0.026085
GO_REGULATION_OF_RESPONSE_TO_CYTOKINE_STIMULUS	149	0.415541	1.693062	0	0.02618
GO_POSITIVE_REGULATION_OF_CELLULAR_COMPONENT_MOVEMENT	482	0.380844	1.690928	0	0.026713
GO_POSITIVE_REGULATION_OF_ALCOHOL_BIOSYNTHETIC_PROCESS	24	0.556706	1.689464	0.013928	0.027058
GO_CALCIUM_MEDIATED_SIGNALING	185	0.405889	1.689257	0	0.027063
GO_NEGATIVE_REGULATION_OF_B_CELL_ACTIVATION	32	0.526841	1.688503	0.009472	0.02721
GO_REGULATION_OF_VESICLE_MEDIATED_TRANSPORT	500	0.382064	1.688194	0	0.027234
GO_I_KAPPAB_KINASE_NF_KAPPAB_SIGNALING	243	0.394415	1.688016	0	0.027247
GO_CELL_ADHESION_MEDIATED_BY_INTEGRIN	63	0.463553	1.685468	0.001267	0.027919
GO_RESPONSE_TO_PROTOZOAN	23	0.564215	1.685465	0.007205	0.027852
GO_REGULATION_OF_ANION_TRANSMEMBRANE_TRANSPORT	27	0.544399	1.68475	0.009972	0.027994
GO_NEGATIVE_REGULATION_OF_RESPONSE_TO_EXTERNAL_STIMULUS	343	0.384972	1.683506	0	0.028305
GO_NEGATIVE_REGULATION_OF_CHEMOTAXIS	54	0.472653	1.682918	0.001258	0.028431
GO_HOMEOSTASIS_OF_NUMBER_OF_CELLS	220	0.394772	1.681965	0	0.028681
GO_SNARE_COMPLEX_ASSEMBLY	16	0.609555	1.6819	0.001479	0.028622
GO_PROTEIN_KINASE_B_SIGNALING	231	0.395972	1.681656	0	0.02863

GO_POSITIVE_REGULATION_OF_CYTOKINE_PRODUCTION_INVOLVED_IN_INFLAMMATORY_RESPONSE	20	0.588124	1.681324	0.01059	0.028658
GO_NEGATIVE_REGULATION_OF_CALCIUM_MEDIATED_SIGNALING	25	0.545743	1.680053	0.005502	0.028993
GO_POSITIVE_REGULATION_OF_CALCIUM_ION_TRANSPORT_INTO_CYTOSOL	50	0.478881	1.679168	0.008906	0.02914
GO_ACTIN_FILAMENT_ORGANIZATION	357	0.382455	1.679073	0	0.029102
GO_TOLL_LIKE_RECEPTOR_4_SIGNALING_PATHWAY	31	0.522801	1.678621	0.008499	0.029176
GO_NEGATIVE_REGULATION_OF_CYTOKINE_SECRETION	24	0.556892	1.678169	0.01264	0.029274
GO_REGULATION_OF_MONOCYTE_CHEMOTAXIS	20	0.579278	1.677795	0.012931	0.029316
GO_INACTIVATION_OF_MAPK_ACTIVITY	25	0.551683	1.676631	0.012097	0.029662
GO_OSTEOCLAST_DIFFERENTIATION	88	0.443222	1.675348	0	0.029947
GO_POSITIVE_REGULATION_OF_GTPASE_ACTIVITY	311	0.38628	1.675198	0	0.029931
GO_NEGATIVE_REGULATION_OF_LYMPHOCYTE_APOPTOTIC_PROCESS	28	0.53997	1.674108	0.001418	0.030209
GO_POSITIVE_REGULATION_OF_MYELOID_LEUKOCYTE_MEDIATED_IMMUNITY	30	0.526473	1.673502	0.011189	0.030331
GO_REGULATION_OF_EXOCYTOSIS	204	0.395912	1.671433	0	0.030951
GO_RESPONSE_TO_INTERLEUKIN_12	46	0.488655	1.671179	0.001312	0.030984
GO_POSITIVE_REGULATION_OF_RECEPTOR_SIGNALING_PATHWAY_VIA_STAT	86	0.43348	1.669516	0.003632	0.031516
GO_ASTROCYTE_ACTIVATION	22	0.56314	1.669198	0.00561	0.031578
GO_NECROTIC_CELL_DEATH	47	0.486445	1.668917	0.002551	0.031605
GO_NEGATIVE_REGULATION_OF_SECRETION	165	0.402414	1.668869	0	0.031555
GO_REGULATION_OF_SYSTEMIC_ARTERIAL_BLOOD_PRESSURE_BY_CIRCULATORY_RENIN_ANGIOTENSIN	18	0.591578	1.668411	0.006051	0.031658
GO_POSITIVE_REGULATION_OF_CELL_ADHESION_MEDIATED_BY_INTEGRIN	19	0.597994	1.668068	0.010279	0.031692
GO_POSITIVE_REGULATION_OF_SECRETION	338	0.381607	1.665973	0	0.032353
GO_REGULATION_OF_CYSTEINE_TYPE_ENDOPEPTIDASE_ACTIVITY	208	0.393309	1.664774	0	0.032686

GO_POSITIVE_REGULATION_OF_CYSTEINE_TYPE_ENDOPEPTIDASE_ACTIVITY	130	0.415583	1.66469	0	0.03265
GO_POSITIVE_REGULATION_OF_I_KAPPAB_KINASE_NF_KAPPAB_SIGNALING	165	0.402573	1.664446	0.001095	0.032658
GO_NEGATIVE_REGULATION_OF_RESPONSE_TO_BIOTIC_STIMULUS	86	0.437133	1.664414	0	0.0326
GO_MEMBRANE_PROTEIN_ECTODOMAIN_PROTEOLYSIS	39	0.497121	1.664281	0.009126	0.03257
GO_NEGATIVE_REGULATION_OF_LEUKOCYTE_CHEMOTAXIS	15	0.620413	1.66419	0.010279	0.032524
GO_POSITIVE_REGULATION_OF_CALCIUM_ION_TRANSPORT	108	0.415979	1.661658	0.001127	0.03336
GO_POSITIVE_REGULATION_OF_CELL_SUBSTRATE_ADHESION	113	0.418263	1.660837	0.003444	0.033599
GO_BONE_CELL_DEVELOPMENT	29	0.525172	1.660476	0.001387	0.033632
GO_NEGATIVE_REGULATION_OF_T_CELL_MEDIATED_IMMUNITY	18	0.599531	1.660456	0.008824	0.033564
GO_POSITIVE_REGULATION_OF_REACTIVE_OXYGEN_SPECIES_METABOLIC_PROCESS	90	0.429329	1.657936	0.00119	0.034333
GO_INTERLEUKIN_17_PRODUCTION	32	0.513395	1.657819	0.006803	0.03429
GO_NEGATIVE_REGULATION_OF_AMYLOID_PRECURSOR_PROTEIN_CATABOLIC_PROCESS	16	0.605884	1.657573	0.0162	0.034287
GO_REGULATION_OF_ENDOCYTOSIS	193	0.391862	1.65714	0	0.034372
GO_REGULATION_OF_VASCULAR_ENDOTHELIAL_GROWTH_FACTOR_RECEPTOR_SIGNALING_PATHWAY	25	0.54558	1.655923	0.00672	0.034727
GO_NEGATIVE_REGULATION_OF_PHOSPHORUS_METABOLIC_PROCESS	463	0.373084	1.655864	0	0.034668
GO_LEUKOCYTE_TETHERING_OR_ROLLING	28	0.531551	1.655311	0.005731	0.034815
GO_RESPONSE_TO_PROSTAGLANDIN	31	0.514832	1.65511	0.01105	0.034836
GO_REGULATION_OF_CELL_ADHESION_MEDIATED_BY_INTEGRIN	43	0.485542	1.654325	0.005222	0.035079
GO_RECEPTOR_BIOSYNTHETIC_PROCESS	23	0.552178	1.654093	0.009929	0.035104
GO_MYD88_DEPENDENT_TOLL_LIKE_RECEPTOR_SIGNALING_PATHWAY	33	0.507365	1.652995	0.005556	0.035418
GO_MHC_CLASS_II_BIOSYNTHETIC_PROCESS	16	0.608768	1.651561	0.008772	0.035897
GO_POSITIVE_REGULATION_OF_NITRIC_OXIDE_SYNTHASE_BIOSYNTHETIC_PROCESS	15	0.622977	1.651237	0.014749	0.035917

GO_REGULATION_OF_MONOOXYGENASE_ACTIVITY	55	0.46 2008	1.65 0682	0.00 3741	0.03 6049
GO_POSITIVE_REGULATION_OF_MAPK_CASCADE	473	0.37 1566	1.64 8297	0	0.03 6851
GO_NEGATIVE_REGULATION_OF_INTERLEUKIN_1_PRODUCTION	25	0.54 7708	1.64 8043	0.00 5479	0.03 687
GO_REGULATION_OF_NATURAL_KILLER_CELL_MEDIATED_IMMUNITY	40	0.48 5824	1.64 7406	0.00 6631	0.03 7007
GO_T_HELPER_1_CELL_DIFFERENTIATION	16	0.60 2674	1.64 6881	0.01 4684	0.03 7126
GO_SOMATIC_DIVERSIFICATION_OF_IMMUNOGLOBULINS	55	0.46 1982	1.64 6604	0.00 2506	0.03 7156
GO_MODULATION_BY_HOST_OF_VIRAL_PROCESS	27	0.53 7056	1.64 2171	0.01 0929	0.03 8806
GO_TOLL_LIKE_RECEPTOR_SIGNALING_PATHWAY	129	0.40 8851	1.64 06	0	0.03 9347
GO_NEGATIVE_REGULATION_OF_PHOSPHORYLATION	381	0.37 2142	1.63 9348	0	0.03 9841
GO_POSITIVE_REGULATION_OF_SUPRAMOLECULAR_FIBER_ORGANIZATION	173	0.39 4919	1.63 8718	0.00 1085	0.04 0017
GO_STRESS_FIBER_ASSEMBLY	88	0.42 66	1.63 8237	0.00 2361	0.04 0124
GO_POSITIVE_REGULATION_OF_T_HELPER_1_TYPE_IMMUNE_RESPONSE	19	0.56 9506	1.63 7722	0.01 3025	0.04 022
GO_RUFFLE_ORGANIZATION	44	0.48 2288	1.63 715	0.00 3953	0.04 0331
GO_REGULATION_OF_CALCIUM_ION_TRANSPORT	218	0.38 4434	1.63 6861	0	0.04 0375
GO_NEGATIVE_REGULATION_OF_NATURAL_KILLER_CELL_MEDIATED_IMMUNITY	17	0.59 3246	1.63 551	0.00 8876	0.04 0817
GO_BARBED_END_ACTIN_FILAMENT_CAPPING	21	0.55 6646	1.63 5442	0.01 4306	0.04 0751
GO_PEPTIDYL_TYROSINE_AUTOPHOSPHORYLATION	41	0.47 7945	1.63 465	0.00 9309	0.04 0979
GO_REGULATION_OF_T_CELL_DIFFERENTIATION_IN_THYMUS	23	0.53 5961	1.63 4445	0.00 9459	0.04 1003
GO_MATURE_B_CELL_DIFFERENTIATION_INVOLVED_IN_IMMUNE_RESPONSE	20	0.56 5146	1.63 2438	0.02 1521	0.04 1833
GO_POSITIVE_REGULATION_OF_LEUKOCYTE_APOPTOTIC_PROCESS	24	0.53 9212	1.63 2424	0.00 2833	0.04 1752
GO_POSITIVE_REGULATION_OF_CYTOSOLIC_CALCIUM_ION_CONCENTRATION_INVOLVED_IN_PHOSPHOL IPASE_C_ACTIVATING_G_PROTEIN_COUPLED_SIGNALING_PATHWAY	28	0.52 1946	1.63 1468	0.00 9485	0.04 2031
GO_POSITIVE_REGULATION_OF_PEPTIDYL_TYROSINE_PHOSPHORYLATION	177	0.39 2943	1.63 1226	0	0.04 2033

GO_RESPONSE_TO_INTERLEUKIN_7	27	0.53<br>079	1.63<br>0496	0.01<br>0944	0.04<br>225
GO_POSITIVE_REGULATION_OF_ACTIN_FILAMENT_BUNDLE_ASSEMBLY	55	0.45<br>4771	1.62<br>9424	0.00<br>507	0.04<br>2651
GO_ANIMAL_ORGAN_REGENERATION	68	0.44<br>4222	1.62<br>9071	0.00<br>3695	0.04<br>2711
GO_REGULATION_OF_RELEASE_OF_CYTOCHROME_C_FROM_MITOCHONDRIA	43	0.47<br>6564	1.62<br>8617	0.01<br>0796	0.04<br>2772
GO_ERYTHROCYTE_HOMEOSTASIS	107	0.41<br>565	1.62<br>832	0	0.04<br>2827
GO_EXTRINSIC_APOPTOTIC_SIGNALING_PATHWAY_VIA_DEATH_DOMAIN_RECEPTORS	81	0.43<br>2282	1.62<br>7823	0.00<br>3584	0.04<br>2951
GO_ACTIVATION_OF_INNATE_IMMUNE_RESPONSE	124	0.40<br>5604	1.62<br>6613	0	0.04<br>3412
GO_PROTEIN_LOCALIZATION_TO_CELL_SURFACE	59	0.45<br>572	1.62<br>6095	0.00<br>4914	0.04<br>359
GO_MORPHOGENESIS_OF_A_POLARIZED_EPITHELIUM	137	0.39<br>9705	1.62<br>5817	0.00<br>1133	0.04<br>3636
GO_PLASMA_MEMBRANE_TUBULATION	15	0.59<br>3889	1.62<br>4836	0.01<br>4577	0.04<br>3942
GO_RAS_PROTEIN_SIGNAL_TRANSDUCTION	300	0.37<br>4858	1.62<br>4472	0	0.04<br>4036
GO_PROTEIN_POLYMERIZATION	241	0.38<br>0823	1.62<br>4327	0	0.04<br>402
GO_REGULATION_OF_COAGULATION	81	0.42<br>3824	1.62<br>4065	0.00<br>8343	0.04<br>4064
GO_SOMATIC_RECOMBINATION_OF_IMMUNOGLOBULIN_GENE_SEGMENTS	48	0.46<br>3626	1.62<br>3675	0.00<br>5109	0.04<br>4145
GO_VACUOLAR_ACIDIFICATION	21	0.56<br>2928	1.62<br>2605	0.01<br>2784	0.04<br>4558
GO_MYOBLAST_DIFFERENTIATION	78	0.42<br>4861	1.62<br>2429	0.00<br>2463	0.04<br>4539
GO_POSITIVE_REGULATION_OF_SMALL_GTPASE_MEDIATED_SIGNAL_TRANSDUCTION	68	0.43<br>5288	1.62<br>1286	0.00<br>1235	0.04<br>4975
GO_REGULATION_OF_LEUKOCYTE_ADHESION_TO_VASCULAR_ENDOTHELIAL_CELL	25	0.53<br>1687	1.62<br>0553	0.01<br>3717	0.04<br>5171
GO_POSITIVE_REGULATION_OF_LEUKOCYTE_DEGRANULATION	23	0.54<br>0322	1.62<br>0329	0.01<br>2968	0.04<br>5192
GO_RESPONSE_TO_PROSTAGLANDIN_E	24	0.53<br>8884	1.62<br>0136	0.01<br>983	0.04<br>52
GO_CELL_SUBSTRATE_JUNCTION_ORGANIZATION	91	0.41<br>6934	1.61<br>9856	0.00<br>1167	0.04<br>5228
GO_POSITIVE_REGULATION_OF_STRESS_ACTIVATED_PROTEIN_KINASE_SIGNALING_CASCADE	148	0.40<br>0387	1.61<br>9692	0	0.04<br>5206

GO_POSITIVE_REGULATION_OF_ISOTYPE_SWITCHING	21	0.55<br>2388	1.61<br>9584	0.01<br>7751	0.04<br>5169
GO_REGULATION_OF_MYOBLAST_FUSION	20	0.56<br>3717	1.61<br>9424	0.01<br>3889	0.04<br>5143
GO_REGULATION_OF_LONG_TERM_SYNAPTIC_POTENTIATION	45	0.47<br>5448	1.61<br>8787	0.01<br>3175	0.04<br>5347
GO_ACTIN_FILAMENT_BUNDLE_ORGANIZATION	134	0.39<br>967	1.61<br>7729	0.00<br>225	0.04<br>5784
GO_POSITIVE_REGULATION_OF_INTERLEUKIN_12_PRODUCTION	32	0.50<br>42	1.61<br>7536	0.01<br>0667	0.04<br>5789
GO_REGULATION_OF_REGULATED_SECRETORY_PATHWAY	150	0.39<br>1678	1.61<br>7229	0.00<br>1114	0.04<br>5837
GO_PIGMENT_GRANULE_LOCALIZATION	23	0.54<br>321	1.61<br>7222	0.01<br>2857	0.04<br>5748
GO_NEGATIVE_REGULATION_OF_PHAGOCYTOSIS	16	0.59<br>469	1.61<br>6775	0.01<br>9316	0.04<br>5843
GO_ASTROCYTE_DIFFERENTIATION	78	0.42<br>6806	1.61<br>6457	0.00<br>2427	0.04<br>5876
GO_NEGATIVE_REGULATION_OF_CELL_KILLING	23	0.54<br>5276	1.61<br>618	0.01<br>3986	0.04<br>5923
GO_REGULATION_OF_SUPRAMOLECULAR_FIBER_ORGANIZATION	301	0.37<br>1647	1.61<br>6146	0	0.04<br>5844
GO_JNK_CASCADE	191	0.38<br>2453	1.61<br>5184	0	0.04<br>6188
GO_EXTRINSIC_APOPTOTIC_SIGNALING_PATHWAY	213	0.37<br>6945	1.61<br>4537	0	0.04<br>6421
GO_BLOOD_VESSEL_REMODELING	40	0.47<br>945	1.61<br>4257	0.00<br>8163	0.04<br>6466
GO_REGULATION_OF_RECEPTOR_LOCALIZATION_TO_SYNAPSE	16	0.58<br>9178	1.61<br>4233	0.01<br>3554	0.04<br>6392
GO_ESTABLISHMENT_OF_TISSUE_POLARITY	118	0.40<br>4751	1.61<br>3949	0	0.04<br>6444
GO_REGULATION_OF_GRANULOCYTE_DIFFERENTIATION	15	0.59<br>6164	1.61<br>3565	0.02<br>3438	0.04<br>6538
GO_T_HELPER_17_TYPE_IMMUNE_RESPONSE	29	0.51<br>307	1.61<br>2103	0.00<br>6983	0.04<br>7119
GO_REGULATION_OF_RECEPTOR_SIGNALING_PATHWAY_VIA_STAT	124	0.40<br>3096	1.61<br>0973	0.00<br>2301	0.04<br>7569
GO_POSITIVE_REGULATION_OF_OXIDOREDUCTASE_ACTIVITY	51	0.45<br>6444	1.60<br>9698	0.01<br>2723	0.04<br>8116
GO_OSTEOCLAST_DEVELOPMENT	16	0.59<br>0299	1.60<br>9647	0.01<br>7241	0.04<br>8039
GO_REGULATION_OF_PROTEIN_LOCALIZATION_TO_CELL_PERIPHERY	106	0.41<br>0238	1.60<br>711	0.00<br>4577	0.04<br>9188

GO_POSITIVE_REGULATION_OF_CELL_MATRIX_ADHESION	53	0.445713	1.606196	0.006361	0.049574
GO_NEGATIVE_REGULATION_OF_PROTEIN_KINASE_B_SIGNALING	37	0.486623	1.605935	0.010695	0.049602
GO_POSITIVE_REGULATION_OF_HUMORAL_IMMUNE_RESPONSE	16	0.584994	1.605005	0.024768	0.049962
GO_PATTERN_RECOGNITION_RECEPTOR_SIGNALING_PATHWAY	163	0.385797	1.604903	0.001101	0.049915
GO_POSITIVE_REGULATION_OF_ACTIVATED_T_CELL_PROLIFERATION	23	0.539412	1.604615	0.014306	0.049963
GO_NEGATIVE_REGULATION_OF_ACTIN_FILAMENT_POLYMERIZATION	50	0.454303	1.604327	0.007742	0.049987
GO_COTRANSLATIONAL_PROTEIN_TARGETING_TO_MEMBRANE	88	-0.59986	-2.8476	0	0
GO_ESTABLISHMENT_OF_PROTEIN_LOCALIZATION_TO_ENDOPLASMIC_RETICULUM	101	-0.53119	-2.57588	0	0
GO_PROTEIN_LOCALIZATION_TO_ENDOPLASMIC_RETICULUM	124	-0.48205	-2.46061	0	0
GO_REPLACEMENT_OSSIFICATION	26	-0.66779	-2.40219	0	2.41E-04
GO_TRANSLATIONAL_INITIATION	152	-0.44169	-2.30945	0	0.001961
GO_DETOXIFICATION_OF_INORGANIC_COMPOUND	15	-0.71434	-2.18396	0	0.008813
GO_NUCLEAR_TRANSCRIBED_MRNA_CATABOLIC_PROCESS_NONSENSE_MEDIATED_DECAY	104	-0.44013	-2.13123	0	0.016007
GO_REGULATION_OF_CHOLESTEROL_METABOLIC_PROCESS	55	-0.46674	-2.08141	0	0.024006
GO_SENSORY_PERCEPTION_OF_SMELL	130	-0.40591	-2.07276	0	0.022832
GO_ENDOCHONDRAL_BONE_MORPHOGENESIS	49	-0.48995	-2.05885	0	0.024115
GO_CYTOPLASMIC_TRANSLATION	68	-0.45	-2.05	0	0.022616

		07	482			
