# Supplementary material for: Construction and Validation of a Macrophage-Associated Risk Model for Predicting the Prognosis of Osteosarcoma
Source: J Oncol. 2021 Jun 2;2021:9967954. doi: 10.1155/2021/9967954 (PMC8192206; doi:10.1155/2021/9967954)
Supplement: Supplementary Materials — Table S1. Full list of 384 macrophage-associated genes. Table S2. Enrichment analysis in TARGET-OS. [file 9967954.f1.zip › 9967954.f1/TableS1.pdf]

Table S1 Full list of MAGs

ABCC6
ABHD16A
ABI3
ACOT12
ADAM21
ADORA2B
AFG3L2
AK3
AK8
AKAP10
AKAP11
AKNAD1
ALOX5AP
ANAPC10
ANGPTL7
ANXA2
APOBEC2
APOBEC3B
APOBEC4
ARHGEF37
ARL5A
ARPP21
ASAH2
ASB13
ASPA
ATP1B1
ATP6V0D1
ATP6V1E1
AVP
B9D2
BAIAP3
BAMBI
BATF2
BBS2
BCAS4
BEX4
BEX5
BFSP2-AS1
BRWD1
C10orf90
C16orf54
C1R
C6
CACNA1A
CACUL1
CADM2
CAMKK1
CAMP
CAPN5
CAPZB
CBY1
CCDC171
CCDC51
CCDC85B
CCL20
CCL5
CCR1

CCT6B
CD84
CDK4
CENPJ
CENPO
CEPT1
CGREF1
CHMP5
CHN1
CHRNA5
CHST11
CHST7
CIB3
CIC
CLCF1
CLDN11
CLEC7A
CLIP3
CLSPN
CMC2
CNOT3
COL12A1
CRYZ
CTSB
CTSG
CXCR5
CXorf38
CYLC2
CYSLTR1
DAO
DDX6
DENND4C
DHRS1
DHX9
DIAPH3
DICER1
DIO2
DMD
DNAJA3
DOCK3
DPM3
DPY19L4
DSP
DUSP23
DYNC1H1
DYRK2
ECT2
EGLN3
EGR3
EHHADH
ELFN2
ENC1
EOMES
EPCAM
FAM110C
FAM126A
FAM160B2
FAM171A1

FAM78A
FAM98B
FAS
FAT1
FBXO31
FBXO46
FCER2
FCRL1
FKBP9
FNTB
FOSL2
FOXG1
FOXRED2
FSD1L
FUBP1
FZD7
GAN
GDF9
GLCE
GLIPR1L1
GNB4
GNPDA2
GNRH1
GOT1
GPN2
GPX1
GRIA2
GYG1
HABP2
HAT1
HCP5
HEATR6
HEBP1
HLA-DQA1
HMGB3P1
HMGN2P46
HOOK2
HP1BP3
HPS3
HSP90AB1
HSPA12B
ICOS
IDH3G
IFI27
IFNA5
IGFBP3
IL16
IL17A
IL1R1
IL6
INTS9
IPO5
IQGAP2
IRAK1
IRS1
ITCH
ITGA4
ITPKB

JUNB
KCNJ16
KCNJ9
KLF10
KLHL10
KLHL9
KRBA1
KRTAP4-3
LAYN
LIMK1
LMNB1
LMNTD2
LRRC3B
LRRC56
MACF1
MAD1L1
MAMDC4
MAN1C1
MAOA
MAP1LC3A
MAP3K5
MAPRE2
MECP2
MED13L
MEF2D
METTL1
METTL21A
MGAT1
MIOS
MOB1A
MOB2
MSN
MT2A
MX1
MYB
MYH9
MYL4
MYOF
NCAPG
NCAPG2
NCF4
NDUFA3
NDUFA4
NDUFB4
NDUFB6
NELFCD
NEUROG1
NFE2
NFKB1
NFKBIE
NIFK
NIPBL
NLGN3
NOL12
NOP16
NPL
NQO2
NR4A1

NT5E
NUBP2
NUDT5
OSGEP
OSMR
OTUB2
OTUD7B
P2RX4
PACRG
PAK1
PATZ1
PCDH12
PCM1
PCOLCE2
PDCD4-AS1
PDGFC
PFDN1
PGK1
PHF8
PHLPP1
PIM1
PITPNA
PKDREJ
PLA2G6
PLAT
PLEKHG3
PLEKHO1
PML
PODXL
POFUT2
PRAM1
PRCP
PRDM9
PRKCZ
PRPF6
PSMB1
PSMB9
PSTK
PTPRCAP
PUS10
QPCT
QSOX1
RAB37
RAD21
RAN
RAP1GDS1
RENBP
RGS1
RIOK3
RNF113A
RPLP1
RRAGC
RREB1
RWDD2A
S100PBP
SBF1
SCCPDH
SCFD2

SDHAF2
SEC61B
SEMA4B
SESTD1
SH2D5
SH3TC1
SH3YL1
SKA1
SLC12A3
SLC16A5
SLC16A6
SLC17A3
SLC1A5
SLC22A18AS
SLC25A51
SLITRK1
SMAP1
SMARCB1
SMPDL3B
SNRPF
SNX21
SNX9
SPATA5L1
SPC25
SPG21
SPNS2
SPSB1
SPTAN1
SPTLC2
SREBF1
SRP72
SSBP4
SSH2
STARD5
SUPV3L1
SURF1
SV2A
SYT1
TAF4B
TAF9
TATDN2
TBC1D22A
TBC1D9B
TDRD3
TEX43
TIMM8B
TLN2
TMED9
TMEM181
TMEM229B
TMEM26
TMEM33
TMEM39B
TMEM50B
TMX3
TMX4
TNFRSF9
TOB1

TOP2B
TPCN1
TPRA1
TRIM26
TRIM45
TRIM59
TRMT61B
TRRAP
TSG101
TSHZ3
TTF1
TXNIP
TXNL1
TYRO3
UBASH3B
UBN1
UBXN1
UCHL3
UIMC1
VAC14
VPS54
VRK2
VSTM2L
WAS
WDFY3
WDFY4
WDR1
WDSUB1
WIPI2
WNT9A
YTHDC2
ZC3H18
ZC3HAV1
ZDHHC20
ZNF195
ZNF214
ZNF706
